# Saliva-based detection of COVID-19 infection in a real-world setting using reagent-free Raman spectroscopy and machine learning

**DOI:** 10.1117/1.JBO.27.2.025002

**Published:** 2022-02-09

**Authors:** Katherine Ember, François Daoust, Myriam Mahfoud, Frédérick Dallaire, Esmat Zamani Ahmad, Trang Tran, Arthur Plante, Mame-Kany Diop, Tien Nguyen, Amélie St-Georges-Robillard, Nassim Ksantini, Julie Lanthier, Antoine Filiatrault, Guillaume Sheehy, Gabriel Beaudoin, Caroline Quach, Dominique Trudel, Frédéric Leblond

**Affiliations:** a Polytechnique Montréal, Montreal, Canada; b Center de recherche du Center hospitalier de l’Université de Montréal, Montreal, Canada; c Institut du cancer de Montréal, Montreal, Canada; d Research Center, CHU Sainte-Justine, Montreal, Canada; e University of Montreal, Faculty of Medicine, Montreal, Quebec, Canada; f Université de Montréal, Department of Pathology and Cellular Biology, Montreal, Quebec, Canada; g Center Hospitalier de l’Université de Montréal, Department of Pathology, Montreal, Quebec, Canada

**Keywords:** coronavirus disease-19, Raman spectroscopy, biofluids, saliva, screening

## Abstract

**Significance:**

The primary method of COVID-19 detection is reverse transcription polymerase chain reaction (RT-PCR) testing. PCR test sensitivity may decrease as more variants of concern arise and reagents may become less specific to the virus.

**Aim:**

We aimed to develop a reagent-free way to detect COVID-19 in a real-world setting with minimal constraints on sample acquisition. The machine learning (ML) models involved could be frequently updated to include spectral information about variants without needing to develop new reagents.

**Approach:**

We present a workflow for collecting, preparing, and imaging dried saliva supernatant droplets using a non-invasive, label-free technique—Raman spectroscopy—to detect changes in the molecular profile of saliva associated with COVID-19 infection.

**Results:**

We used an innovative multiple instance learning-based ML approach and droplet segmentation to analyze droplets. Amongst all confounding factors, we discriminated between COVID-positive and COVID-negative individuals yielding receiver operating coefficient curves with an area under curve (AUC) of 0.8 in both males (79% sensitivity and 75% specificity) and females (84% sensitivity and 64% specificity). Taking the sex of the saliva donor into account increased the AUC by 5%.

**Conclusion:**

These findings may pave the way for new rapid Raman spectroscopic screening tools for COVID-19 and other infectious diseases.

## 1 Introduction

### 1.1 Current State of the COVID-19 Pandemic

COVID-19 has precipitated the deaths of 3.9 million people worldwide as of the end of June 2021 and is the world’s most costly health crisis to date. The cost of the pandemic to the United States economy is projected to amount to $16 trillion.^1^ In a 19-month period, the severe acute respiratory syndrome (SARS)-coronavirus 2 (CoV-2) virus has infected over 182 million people and numbers of confirmed cases continue to grow.^2^ Key difficulties in controlling the spread of SARS-CoV-2 are the long pre-symptomatic period (median period of 5 days^3^), the wide range of non-specific symptoms (such as, coughing, sneezing, fever, and headaches),^4^ and the fact that asymptomatic individuals may be contagious.^5^ As governments worldwide have issued lockdowns and travel bans, rapid viral screening has become a crucial method to limit the spread of SARS-CoV-2.

### 1.2 RT-PCR as a Gold Standard

The current gold standards for SARS-CoV-2 testing are nucleic acid amplification tests (NAATs) using saliva or oro-nasopharyngeal swabs in which viral ribonucleic acid (RNA) is amplified and detected using tools, such as reverse transcription polymerase chain reaction (RT-PCR).^6^ This uses nucleic acid primers, enzymes, and cycles of heat to amplify a specific genomic sequence (from the SARS-CoV-2 genome in this case), enabling it to be detected more easily.^7^ The sensitivity and specificity for saliva-based NAATs are ∼83% and 99%, respectively, and the sensitivity and specificity for nasopharyngeal swab-based NAATs are ∼84% and 99%, respectively.^6^ Despite the fact that RT-PCR has successfully been used in testing for respiratory diseases, this method can show lower sensitivity for SARS-CoV-2 detection before presentation of symptoms.^8^ Identifying asymptomatic patients early on can help prevent and control the spread of COVID-19, so RT-PCR may fall short as a tool for mass serial screening of asymptomatic populations.^7^^,^^9^ Additionally, this method of testing typically necessitates time-consuming transport of samples to clinical laboratories where complex tailored reagents are used.^10^ These reagents may be in short supply and lose specificity as the virus mutates, which has already been shown to be the case with influenza.^11^ To date, the Center for Disease Control has identified seven variants of concern or variants of interest^12^ and more will undoubtedly arise. We need to investigate reagent-free, on-site, rapid screening approaches to reliably detect cases to control outbreaks and limit community spread of the disease.^13^

### 1.3 Saliva as a Biosample

There are two primary ways of collecting SARS-CoV-2 RNA from a patient: nasopharyngeal (NPG) swabs and saliva sampling.^6^ NPG swabs are invasive and uncomfortable. There is also an increased risk for healthcare workers to be exposed to viruses due to patients sneezing or coughing.^14^ Meanwhile, saliva can be self-collected using non-invasive means, making it a much more attractive option for frequent screening. Saliva has been used successfully as a diagnostic tool for other coronavirus infections as the oral and nasal cavities can act as points of entry for respiratory viruses.^15^ SARS-CoV-2 may also enter the saliva from debris of the NPG epithelium, which drains into the oral cavity or from infection of the salivary glands.^16^ Consequently, using saliva samples paired with a reagent-free alternative approach could be an option for COVID-19 detection.

### 1.4 Raman Spectroscopy

Raman spectroscopy is a method of assessing the molecular composition of samples in a reagent-free manner. It is a light-based technique, which measures the inelastic light scattered by matter, also called Raman scattering.^17^ This phenomenon was predicted in 1928 by Smekal^18^ and observed experimentally in 1928 by Raman and Krishnan,^19^ but the potential biomedical application of Raman spectroscopy did not emerge until 1970.^20^ Raman scattering occurs when there is an exchange of energy between a sample and a monochromatic laser source emitting either visible or near-infrared light. The exchange of energy results in photons of scattered light with wavelengths shifted compared to the excitation source in a way that depends on molecular structure and bonding. The difference in wavelength of the Raman scattered light compared to the incident light is called the Raman shift, and two different effects can be observed: the Stokes shift (red shift) or anti-Stokes shift (blue shift).^17^^,^^21^ The Raman shift is usually measured in wavenumbers (${\mathrm{cm}}^{-1}$), which are units that are inversely proportional to wavelength. Since the ground state is more populated at thermal equilibrium, the Stokes shift is more prevalent and commonly used in Raman spectroscopy.^21^^,^^22^

In a Raman spectrometer, a detector is used to measure light that has been inelastically scattered from a sample after it has passed through a series of filters, and these measurements are converted into a Raman spectrum. This is a plot of the intensity of the scattered light against the Raman shift in wavenumbers.^23^ Intensity of a Raman peak at a particular Raman shift increases as the concentration of molecules responsible for the peak increases. Therefore, a Raman spectrum can be thought of as a molecular fingerprint giving information about the molecules present in the sample through the analysis of the position, height, and width of peaks present in the spectrum. Raman spectroscopy can identify biomolecular features (such as lipids, proteins, nucleic acids, and amino acids) within biological samples and discriminate between organs, tissues, and biofluids based on disease state. In recent years, clinical applications of this light-based technique have gained traction in oncology,^24^^–^^26^ inflammatory diseases,^27^^,^^28^ transplantation,^29^ and virology.^30^^,^^31^ For example, Camacho et al.^30^ developed surface enhanced Raman sensors for the detection of the Zika virus by functionalizing core-shell nanoparticles with Zika ZIKV NS1 antibodies and reported a sensitivity of 10  ng/ml.

Machine learning (ML) techniques can be used to classify biological samples based on their Raman spectra. Typically, Raman spectra from a single sample are averaged or are individually labeled before supervised ML is applied. However, this does not allow for spectral heterogeneity within a sample (e.g., due to heterogeneous spatial distribution of molecules). Multiple instance learning (MIL) could provide a method for analyzing heterogeneous samples, e.g., dried droplets. Instead of labeling each individual spectrum, multiple spectra from a single sample are grouped into a “bag”, which is then labeled.^32^^,^^33^

### 1.5 Metabolic Changes

As the concentration of analytes is correlated with the intensity, width, and specificity of Raman peaks, Raman spectroscopy can be used to detect changes in concentration of any Raman-active molecules, e.g., proteins, lipids, and small metabolites.^34^ While not all metabolic impacts of SARS-CoV-2 are known, some have been identified^35^ or can be anticipated from diseases with similar mechanisms of action. The SARS virus, SARS-CoV-1, has been shown to induce long-term metabolic changes in formerly infected patients.^36^ Studies of serum from COVID-positive versus COVID-negative individuals show changes in lipid and tryptophan metabolism,^35^ and membrane-bound mucin type I and secreted mucin type 5A have been found to be elevated in the airways of COVID patients.^37^ The transmission vectors of the SARS-CoV-2 virus have been identified through the propagation of aerosols of biofluids, such as saliva carrying a viral load.^38^^,^^39^ ACE2 is a receptor involved in the mechanism of entry of the virus into cells, and there is a high expression of angiotensin-converting enzyme II (ACE2) in epithelial cells of the oral cavity so it may be possible to detect the virus itself.^40^ Furthermore, it has been hypothesized that COVID-19 may cause bacterial infection and other diseases of the salivary gland, which may in turn bring about changes in molecules within saliva.^41^

### 1.6 Point-of-Care Rapid Screening

In a pandemic context, fast near real-time screening is required to track and contain the propagation of a virus. Airports, schools, workplaces, and remote communities would benefit from a rapid, reagent-free screening technique. The sensitivity and the label-free approach of Raman spectroscopy makes it a candidate for fast, robust, low-cost, and transportable means of viral screening to complement the diagnostic capacity of the gold standards. Any COVID screening tool should be applicable to all users, regardless of symptoms, age, sex, time of sample collection, or diet. This poses a challenge when developing a label-free Raman spectroscopic approach as saliva composition is affected by time of day, sex, age, and potentially other underlying health conditions.^42^^–^^48^

A 2021 study (n=30 for COVID positive volunteers) found that Raman micro-spectroscopy could detect COVID-19 infection in saliva with 84% sensitivity and 92% specificity.^49^ However, the clinical cohort was restricted to elderly volunteers who had presented to hospitals, and the study design required saliva collection prior to breakfast. About 36% of positive cases were severely or critically ill, and a further 30% had symptoms and evidence of pneumonia upon imaging. Another recent study (n=29 for COVID positive volunteers) determined infrared spectroscopy could be used to discriminate between COVID-infected and non-infected saliva with 93% sensitivity and 82% specificity.^50^ As for the previous study, both the COVID-positive and COVID-negative cohorts consisted of hospitalized, symptomatic patients requiring treatment. Most label-free tests would be aimed at screening non-hospitalized people who may be symptomatic or asymptomatic with different levels of severity of COVID-19 infection. Sample collection information and other clinical characteristics apart from viral load were not reported for this dataset, including age, sex, and comorbidities. To be applicable in a setting outside of hospitals, a label-free screening technique must be applicable to people of all ages, with or without symptoms, regardless of diets, smoking status, with samples collected at any time of day.

Other label-free analytical techniques include mass spectrometry (MS) and nuclear magnetic resonance (NMR) spectroscopy. An MS-based technique for COVID-19 has been developed, however, this requires a specific protein to be used to capture the virus and samples must be transported to a testing lab.^51^ NMR spectrometers are typically large, costly, and require trained personnel. Although benchtop spectrometers are available,^52^ there have yet to be any studies applying these to COVID-19.

Here, we present the results of a study demonstrating that Raman spectroscopy combined with ML is a candidate for real-world COVID-19 screening in the general population. The study design was developed to ensure that the number of samples collected allowed us to match COVID-positive and COVID-negative samples in terms of potential confounding factors including sex at birth, age, COVID symptoms, body mass index (BMI), and prescription drugs taken. We studied saliva samples taken from volunteers at a COVID-19 testing clinic, including asymptomatic volunteers and those with respiratory and non-respiratory symptoms. Samples were taken from people aged >10 years old to <61 years old. We found that we could detect COVID-19 infected saliva with 79% sensitivity and 75% specificity in males, and 84% sensitivity and 64% specificity in females. This ML-based technique could be adjusted to increase sensitivity and reduce specificity (or vice versa) where required. It is also the first published application of multiple instance learning either via embedded instance selection (MILES) or discriminative mapping (MILDM) to Raman spectral data, allowing us to account for different molecular content of Raman spectra acquired from the same sample.

## 2 Experimental

### 2.1 Sample Collection

**Fig. 1:**
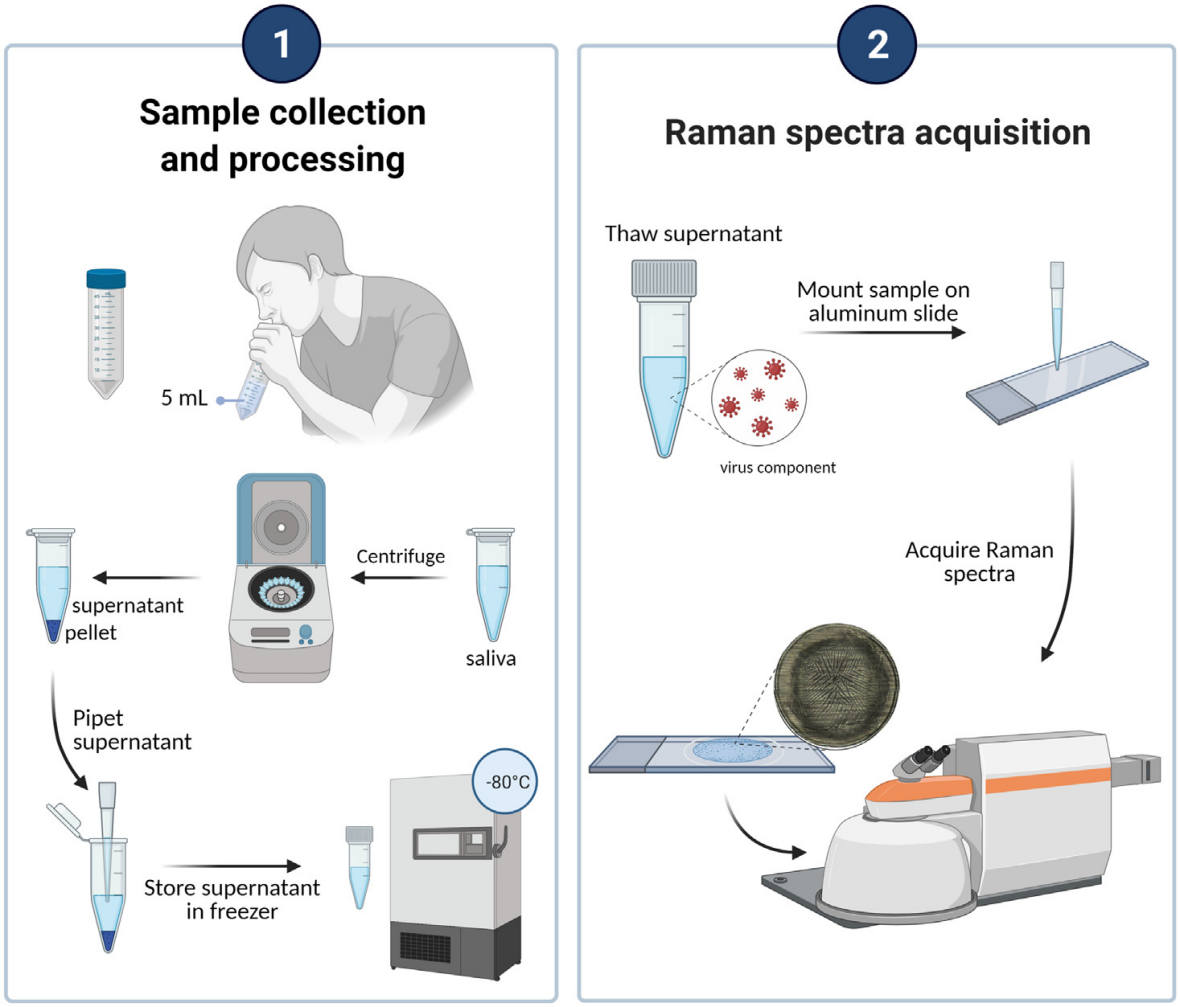
Workflow from saliva collection to determination of COVID infection status from Raman spectra. Volunteers donated between 1 and 5 ml of saliva into a 50-ml tube. The liquid was pipetted into a 1.5-ml microcentrifuge tube which was centrifuged. The saliva supernatant and pellet were then stored separately at −80°C but supernatant only is shown here for clarity. Supernatant was thawed, vortexed and mounted on an aluminum slide. After 45 min of drying, spectra were acquired using a Renishaw InVia Raman spectrometer. This figure was created with BioRender.com.

The experimental workflow is shown in Fig. 1. A total of 37 COVID-19 positive and 513 COVID-19 negative samples were collected from the Pointe-Saint-Charles COVID-19 testing clinic in accordance with ethical guidelines from the Centre Hospitalier de l’Université de Montréal (CHUM) Research Ethics Board (project number: 20.133). Volunteers presenting between 10 am and 2 pm were asked to complete a questionnaire reporting their symptoms and associated biological and environmental factors that could affect saliva composition, e.g., age, sex at birth, and comorbidities. When applicable, volunteers were asked to remove any lipstick or lip balm using makeup removal wipes (About Face Cleansing Wipes, Micronova Manufacturing Inc., Torrance, California). Each volunteer was instructed to first rinse their mouth three times with bottled water to remove food debris. Water has very weak Raman signal. Volunteers then waited 5 to 10 min for saliva to accumulate before spitting in a 50-ml Falcon tube to collect a minimum of 1.5 ml of the biofluid. Tubes were then immediately stored in a refrigerator at 4°C. Samples were transported to the Centre de Recherche du CHUM (CRCHUM) on ice. Samples were classified as being from asymptomatic, non-respiratory symptomatic, or symptomatic volunteers based on symptoms reported in the questionnaire. “Respiratory” symptoms included those that might result in significant amounts of mucus in saliva, such as runny nose, difficulty breathing, sore throat, coughing, or wheezing. “Non-respiratory” symptoms included all other symptoms including headache, muscle aches, and tiredness. The classification of each sample as being obtained from a COVID-19 positive or negative volunteer was based on PCR tests based on either NPG swabs or saliva tests, depending on test availability. Meta-analysis by Butler-Laporte et al.^6^ demonstrates sensitivity of saliva-based tests to be ∼83%, and sensitivity of NPG swabs to be ∼84%. Specificity of both is ∼99%.

### 2.2 Pre-Imaging Sample Processing Protocol

Saliva samples were processed and imaged at biosafety containment level 2 (BSL2) in biosafety cabinets. About 1 ml of whole saliva samples were transferred to 1.5-ml microcentrifuge tubes and centrifuged at 4000 rpm for 30 min at 4°C. The supernatant was pipetted into one cryotube, mixed, and then 40 to 500  μl were aliquoted into five separate 1.8-ml cryotubes. The part of the supernatant near the pellet was discarded, and the pellet was retained in the microcentrifuge tube. Pellet and supernatant samples were stored at −80°C. Prior to Raman spectroscopy interrogation, saliva supernatant samples were thawed at room temperature for 30 min, vortexed for 40 s, and 10  μl were pipetted onto an aluminum slide. Droplets were allowed to dry at room temperature for at least 45 min.

### 2.3 Model Saliva Preparation

To ensure we could relate morphology of dried droplets of saliva to major contributors to the Raman signal and to assign major peaks, we took spectra from dried model saliva. This was made using concentrations listed in Table S2 in the Supplementary Material (adapted from Sarkar et al.^53^). As for human saliva supernatant, it was frozen at −80°C and thawed at room temperature for 30 min, vortexed for 40 s and 10  μl of sample were pipetted onto an aluminum slide and dried. Bovine submaxillary mucin (Sigma Aldrich, St. Louis, Missouri, product A2153) was used to generate the model saliva due to limitations in availability of human mucin. To address this limitation, Raman spectra from a limited available quantity of human mucin type I (Sino Biologic, Beijing, China, product 12123-HCCH) were also acquired for more accurate human mucin peak assignment in human saliva supernatant samples. As mentioned in Sec. 1.5, this type of mucin has been found to be elevated in the airways of COVID patients. For other biomolecules not present in our recipe such as lipids and nucleic acids, we assigned peaks from literature values in Table S3 in the Supplementary Material.

### 2.4 Raman Microspectroscopy of Dried Saliva Samples

Dried saliva supernatant samples and model saliva were imaged using an inVia™ confocal Raman microscope (Renishaw, Gloucester) in reflection mode. Brightfield montage images of each droplet were obtained with a 5× lens (N PLAN, numerical aperture=0.12, air immersion). Brightfield montage images of the central crystalline region and edge region were obtained using a 50× long working distance objective (N PLAN, numerical aperture=0.50, air immersion). For Raman spectral acquisitions, the excitation consisted of a 785-nm 40-mW laser in line-focus mode (3  μm×8  μm spot size) with a 1200  l/mm grating. A dual set of matched dielectric edge filters (785-nm Rayleigh edge filters) were used to remove the laser light. Spectra were acquired in the fingerprint region (between 602 and $1726\;{\mathrm{cm}}^{-1}$) (Fig. 4) from three separate morphological regions within each droplet: “edge” (the perimeter of the droplet), “on crystal” (inside visible crystals in the center of the droplet), and “off crystal” (outside visible crystals in the center of the droplet). The fingerprint region is composed of peaks due to a broad range of molecular vibrations (e.g., C=C, C–N, and P=O), any of which could be perturbed in a disease state. Ten spectra were acquired in each region and 8 to 10 repeat measurements were taken from each point. Spectra were taken from random on crystal and off crystal regions in the densest region of the droplet, and in a zig–zag pattern within the edge to capture spectra from the very edge and slightly closer to the center. Each measurement was made ensuring 60% to 70% of the Raman microscope sensor dynamical range was used to minimize the impact of shot noise, resulting in an acquisition time of 2 to 10 s for edge and 15 to 40 s for center spectra (on crystal and off crystal), depending on the level of sample autofluorescence. Wire 4.4 by Renishaw was used to visualise the data and compile the brightfield image of the sample.

### 2.5 Raman Microspectroscopy of Individual Model Saliva Components

Solid compounds placed on aluminum slides were imaged using the Renishaw InVia microscope. About 25 spectra were taken from each compound using the 50× lens between 105 and $\text{1725 }{\mathrm{cm}}^{-1}$, and 2601 and $3359\;{\mathrm{cm}}^{-1}$.

### 2.6 Spectral Data Processing

The following data pre-processing steps were applied to every individual measurement: (1) removal of cosmic rays with an in-house algorithm using the first derivative to detect narrow peaks ($∼2\;{\mathrm{cm}}^{-1}$) with very large intensities; (2) smoothing using a Savitzky–Golay filter^54^ of order 3 with a window size of 11; (3) background subtraction of signals produced by the aluminum slides and autofluorescence using a custom adaptation of the rolling ball algorithm;^55^ (4) cropping the region below $1100\;{\mathrm{cm}}^{-1}$ due to the large variances at lower wavenumber shifts (likely due to wide ranges in healthy salivary salt concentrations);^56^ (5) averaging of the repeat measurements taken at a given spatial point; and (6) standard normal variate (SNV) normalization.^26^

### 2.7 Data Preparation and Feature Selection

**Fig. 2:**
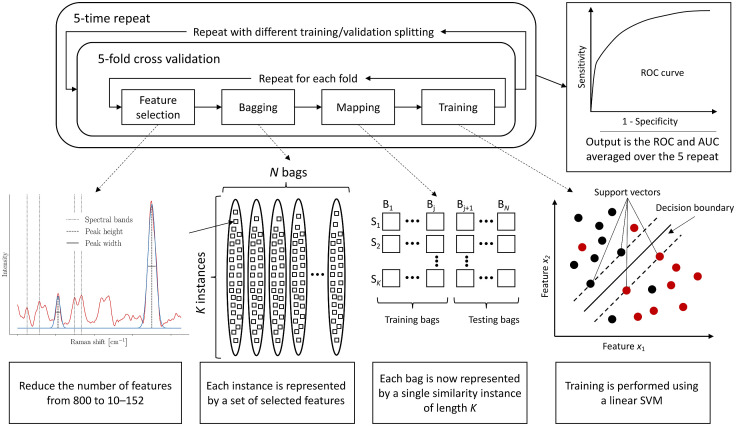
ML schematic workflow. The ML workflow consists of a 5-fold CV embedded in a 5-time repeat loop creating different splitting of the training and validation sets. A 5-time repeat 5-fold CV allowed assessment of variance in AUCs produced by the models, thus reducing the bias induced when splitting the dataset while allowing computation time to remain reasonable. AUCs were stable using this procedure. Feature selection, bagging, mapping, and training steps are repeated for each fold. Raman spectra are represented by spectral peaks and fitted peaks. Each instance is represented by the relevant features which are selected using a combination of a variance-based algorithm, acting as a broad skimmer, and an RF. Each bag is then mapped from a multiple instance representation to a single instance representation through an instance similarity measure; the mapping function being different for MILES and MILDM. A linear SVM algorithm is used to train each model and output a classification probability for patients in the validation set. After each CV procedure, a receiver operating ROC curve is computed with a corresponding AUC value to assess the model performance. The final output is the ROC and AUC averaged over the five repetitions, ensuring further stability. Computational time for each classification scenario was 30 min or less.

For data quality reasons, a limited number of spectra (from three patients for on crystal, from five patients for edge) were removed before training the ML models. Exclusion was based on abnormally high levels of residual stochastic noise (after background removal) or the presence of unusual spectral shapes unrelated to biomolecular content, e.g., incomplete lipstick removal (Fig. S1 in the Supplementary Material). For each spectrum, a Gaussian fitting procedure was applied to each peak of biological origin fitted to extract its position, its height, and its width; a total of 24 peaks were extracted using this procedure. These, along with the relative intensity of 700 individual bands in a spectrum, represent the data from which ML models could be trained (Fig. 2). Standardization of the data, where each feature is individually normalized to exhibit a mean of 0 and unit variance, was performed before the feature selection and classification steps.

A feature selection technique was applied to reduce the size of the feature set prior to model ML model training. Feature reduction is necessary because some spectral regions either provide no useful information for the classification or are perhaps too correlated. Algorithms typically run with at least one parameter, known as a hyperparameter, that needs to be initialized. A variance-based algorithm called SelectKBest^57^ was used to coarsely reduce the number of features below 10% of the total number of features, to speed up the multivariate technique in the next step. Features presenting a large variance across the complete dataset have a higher weight and by sorting them according to their weight, only the k-best features are retained, where k was varied between 5 and 80 for spectral features, and 5 and 72 for peak features. A random forest classifier (RF)^58^ with 200 estimators is then used to further reduce the feature set; a multivariate approach is more robust to prevent selecting highly correlated features. Again, this was done independently for spectral and peak features. In the end, the final number of features will range between 10 and 152, with an average of 90.

### 2.8 Classification Model and Statistical Analyses

A multiple-instance learning (MIL) approach was favored to be at the core of the classification algorithm. In such a scenario, all the spectra (instances) belonging to a given patient are represented by a bag to which a label is associated (COVID positive or COVID negative) rather than labeling the instances individually (Fig. 2). Labels associated with each patient (bag) correspond to the result from their PCR test. More specifically, the selected method was an MIL via embedded instance selection (MILES) algorithm that computes a similarity measure between instances to map them into a different feature space.^32^ Instead of being represented by multiple instances from the original feature, made of spectral and peak features, each bag was represented by a single instance that lay in a new feature space containing the similarity measures. Under MILES, the mapping was done using an intermediate instance pool (IIP)—a matrix that contains all the instances. It was also possible to create a discriminative instance pool (DIP), which is a subset of the IIP represented only by the best instances. In the new feature space, each instance representing a bag had the dimensions of the number of instances forming the IIP or DIP matrix. The method using a DIP is called an MIL with MILDM algorithm.^33^ The similarity function has a hyperparameter, $\sigma^{2}$, that varies between 1 and 70. For high $\sigma^{2}$ values, the similarity function tends to 1, and 0 for small $\sigma^{2}$ values. The size of the DIP is defined by a hyperparameter, $m_{\text{dip}}$, that varies between 5% and 60% of the total number of spectra. Following the mapping, for both MILES and MILDM algorithms, the same feature selection algorithm used for peak and peak features is applied to the new mapped feature space. The number of features in the MILES and MILDM spaces are defined by hyperparameters $k_{\mathrm{MILES}}$ and $k_{\mathrm{MILDM}}$ that vary between 5% and 60% of the total number of spectra and between 5 and $m_{\text{dip}}$, respectively. An RF classifier with 200 estimators is applied subsequently. Once the bags have been mapped to the new feature space, either with MILES or MILDM, a standard support vector machine (SVM) algorithm is used for the training.^59^ Because we used a linear SVM, the only hyperparameter is the regularization parameter C, which corresponds to the penalty term and was varied between 1 and 50.

As the values for the hyperparameters of the feature selection and classification algorithms are not known *a priori*, many combinations need to be considered. A combination is formed by randomly selecting a value for each hyperparameter within their respective range. A 5-fold cross validation (CV) procedure is used to assess the classification performance for each hyperparameter combination. Once applied on the validation set, the trained model will output a classification probability, continuous between 0 and 1, for each sample in the validation set. This procedure is repeated until all folds have been used once as a validation set. The model performance is assessed with a receiver operating characteristic (ROC) curve from which we can compute the accuracy, sensitivity, and specificity associated with the point with the minimal distance to the upper left corner and the area under curve (AUC). The ROC curve is computed by comparing the classification probabilities from the validation sets to their pathological labels, either 0 (COVID negative) or 1 (COVID positive), by varying the threshold value between 0 and 1.

To test the repeatability of the results, all steps were repeated five times, allowing the data to be split differently between the folds. The final performance assessment is an average ROC curve with its own AUC associated. This procedure is repeated until all the desired hyperparameter combinations have been considered and the set of hyperparameters with the highest performance corresponds to the final model.

To assess overfitting of classification models, the steps were repeated using random class labels instead of the class labels associated with the data. The models were run 96 times using random class labels and 96 times using the true labels, and histograms of the AUC generated by each model were plotted.

Data processing, feature selection, and classification models were carried out using Python and the Scikit-learn library.^57^

## 3 Results

### 3.1 Saliva Collection and Preparation Protocol, Volunteer Demographics

We developed a technique for obtaining Raman spectra from saliva supernatant in a way that minimized person-to-person variation (Fig. 1). Raman spectroscopy is sensitive to all Raman-active molecules within a sample and so minimizing exogenous particles is critical. We had previously found that the Raman spectrum of chromophores was present in volunteers who donated saliva while wearing lipstick (Fig. S1 in the Supplementary Material). Therefore, we provided volunteers with lipstick removal wipes if necessary and implemented a mouth washing step to remove food debris. After a waiting time of 5 min, whole saliva was collected from 550 volunteers at a COVID-19 testing site and processed in a containment level 2 facility (clinical characteristics listed in Table 1). Saliva was centrifuged to remove further food particles and the supernatant stored at −80°C. The supernatant was allowed to thaw at room temperature. It was then vortexed, dried on an aluminum slide, and analyzed using a Raman microspectrometer (InVia, Renishaw). Spectra obtained from aqueous phase saliva supernatant were primarily due to fluorescence with no visible Raman peaks, but upon drying we could obtain Raman spectra with clearly distinguishable Raman peaks. The methodology is described in the materials and methods section in more detail. Clinical characteristics of the volunteers are reported in Table 1. The viral loads of our samples ranged from a cycle threshold of 15.5 (very high) to 36.3 (very low) (Table S1 in the Supplementary Material). Not all viral loads were made available to us.

**Table 1:** Clinical characteristics of the total volunteer cohort. Characteristics were taken from questionnaires given to volunteers. There were 513 COVID negative volunteers and 37 COVID positive volunteers. The number on the left in each column is the number of individuals with each characteristic, and the number in parentheses on the right is the percentage of the total number of COVID negative or positive volunteers. About 38 COVID-19 negative and 33 COVID-19 positive samples were analyzed due to time constraints and accessibility to biosafety level 2 containment facilities. Data from the remaining volunteers were not used in this paper but samples have been retained for future studies.

	Total COVID-19 negative	Analyzed COVID-19 negative	Analyzed COVID-19 positive
Total number of volunteers	513	38	33
Age range, n (%)	—	—	—
0–20	58 (11)	6 (15)	7 (21)
21–40	255 (50)	17 (45)	13 (39)
41–60	135 (26)	12 (32)	11 (33)
61–80	60 (12)	3 (8)	2 (6)
81+	1 (0)	0 (0)	0 (0)
Not given	4 (1)	0 (0)	0 (0)
Sex at birth, n (%)	—	—	—
Female	225 (44)	18 (47)	18 (55)
Male	206 (40)	20 (53)	15 (45)
Prefer not to say	82 (16)	0 (0)	0 (0)
Symptoms, n (%)	—	—	—
Respiratory symptoms	187 (36)	24 (61)	21 (64)
Non-respiratory symptoms	30 (6)	1 (3)	3 (9)
None	279 (54)	13 (34)	5 (15)
Not reported	17 (3)	4 (11)	4 (12)
Disease, n (%)	—	—	—
Other disease	124 (24)	10 (26)	3 (9)
None	385 (76)	18 (74)	30 (91)
Nicotine consumption, n (%)	—	—	—
Smoking	96 (19)	4 (11)	3 (9)
Vaping	32 (6)	2 (5)	0 (0)
Alcohol consumption, n (%)	323 (63)	24 (63)	18 (54)
BMI	25.4	27.4	24.6
Prescription medication or vitamins taken	294 (57.3)	27 (71)	24 (73)

### 3.2 Human Saliva Supernatant Forms Morphological Regions with Distinct Raman Spectra

**Fig. 3:**
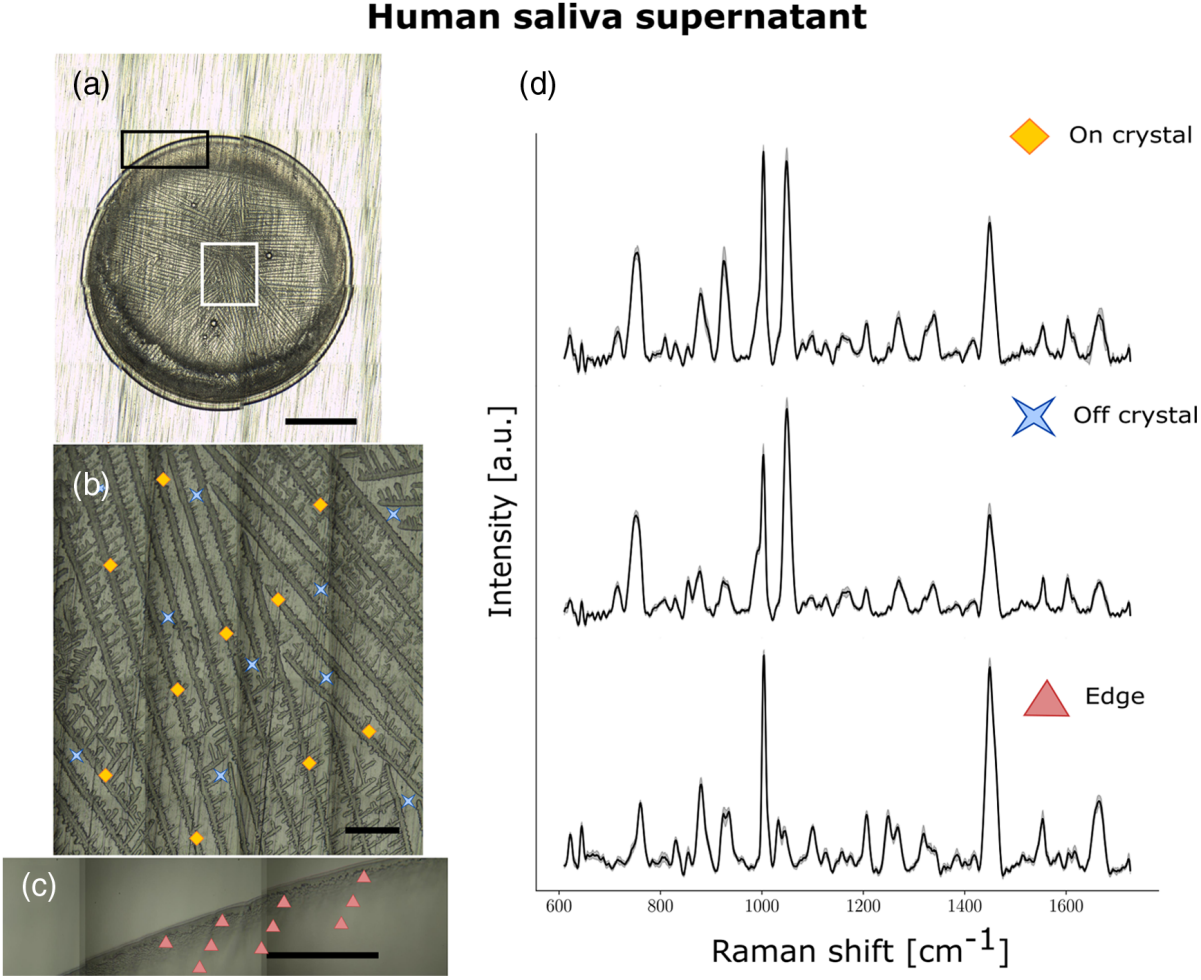
Raman spectroscopy of a representative droplet of human saliva supernatant. (a)–(c) Brightfield images for (a) whole droplet dried on aluminum slide (5×); (b) crystalline region (50×); and (c) edge (50×) with acquisition points shown with different symbols: diamonds (on crystal), crosses (off crystal), and triangles (edge). (d) Average spectra from one saliva sample for on crystal (top), off crystal (middle), and edge (bottom) regions, respectively, with shaded areas representing the interspectral variance within the specimen. Each spectrum is an average of multiple acquisitions obtained with a 785-nm laser using a Renishaw InVia Raman microscope. The scale bar in A is 1 mm in length, whereas the scale bar in B and C are 0.1-mm long.

Raman microspectrometers are spectrometers coupled to microscopes to yield Raman spectra with a spatial resolution that can be modulated by the use of objectives with different magnifications.^60^ A single Raman spectrum can be obtained from a discrete point within a sample and the area of excitation in these experiments was 3  μm by 8  μm, using a 50× objective (N PLAN, numerical aperture=0.50, air immersion). In brightfield images (5× and 50×) taken with the system, we observed that drops of human saliva supernatant most frequently dried with a translucent crystalline region in the center and a slightly more opaque peak around the edge [Figs. 3(a)–3(c)]. This is a common phenomenon observed in dried water-based droplets due to the coffee-ring effect in which suspended particles accumulate at the edge of the droplet due to capillary flow.^61^ In some saliva droplets, there was also a region between the crystalline center and edge where there were no crystals and Raman signal was minimal, which could be attributed to a lower concentration of Raman-active molecules.

To determine which part of the droplet should be interrogated for discrimination between COVID-negative and COVID-positive samples, we took measurements from both the center [Fig. 3(b)] and the edge [Fig. 3(c)] of the dried droplet. The central region was divided into on crystal—where measurements were taken from visible crystals—and off crystal—where measurements were taken from the milieu in-between crystals. Spectra were taken at 10 points in each of the edge, on crystal, and off crystal regions with 8 to 10 successive acquisitions taken from each point to increase signal-to-noise ratio by averaging the spectra [Fig. 3(d)]. In the on crystal and off crystal central regions of human saliva, we observed strong peaks at 1003 and $1045\;{\mathrm{cm}}^{-1}$ as well as 853 and $925\;{\mathrm{cm}}^{-1}$ in some volunteers. In the edge region, we observed strong peaks at 1003, 1449, and $1665\;{\mathrm{cm}}^{-1}$ (Table S3 in the Supplementary Material). These different Raman signatures indicate that the molecular content of the center and the edge of dried saliva supernatant is different. As a droplet dries, certain molecules accumulate at the edge and others in the center. The strong peak at $1003\;{\mathrm{cm}}^{-1}$ is common in Raman studies of biological materials as it corresponds to the ring breathing mode of phenylalanine and is often one of the strongest peak in proteins.^62^

### 3.3 Raman Peak Assignment by Comparison of Human Saliva to Model Saliva

**Fig. 4:**
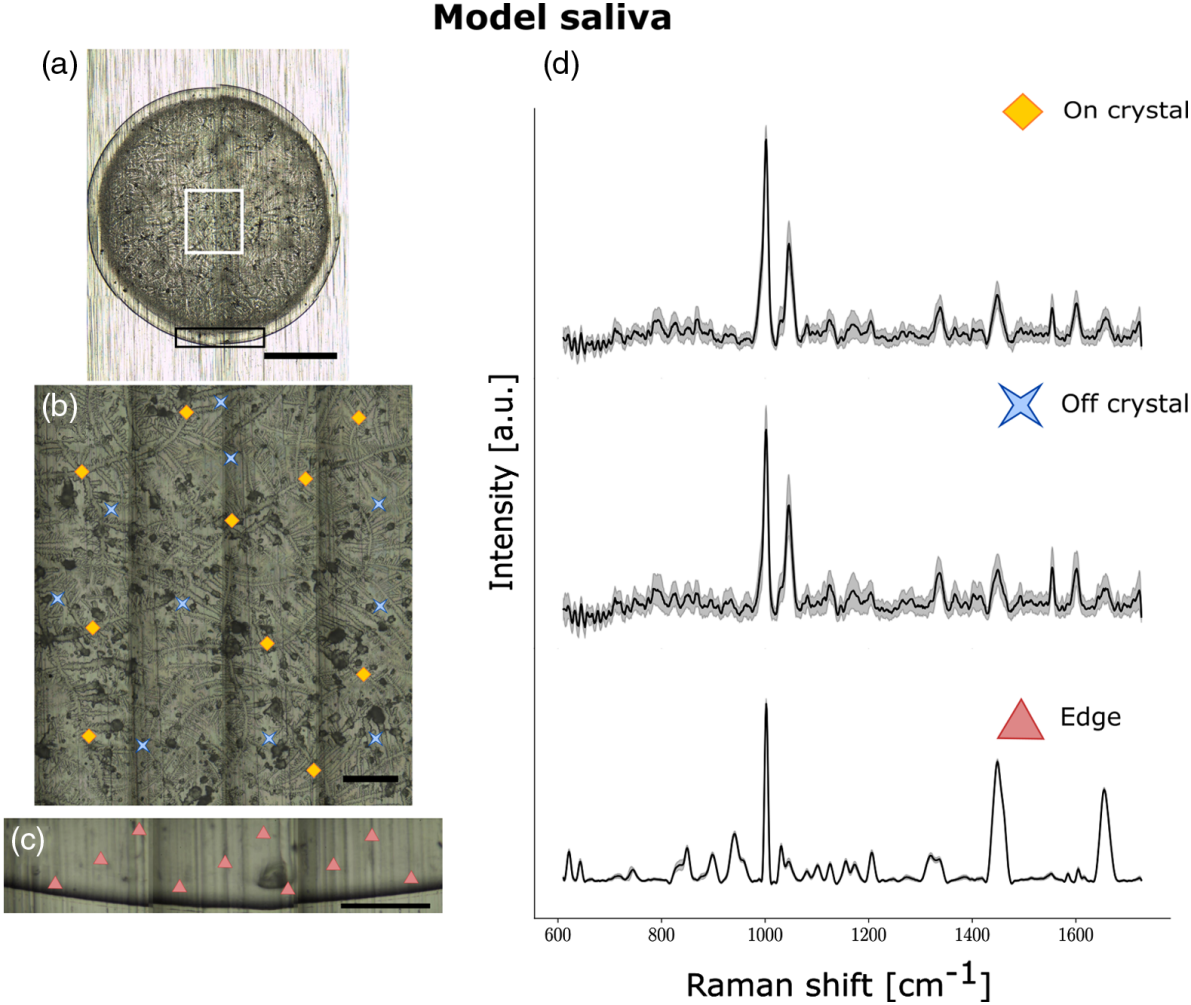
Raman spectroscopy from a droplet of model saliva composed of a mix of salts, bovine serum albumin, mucin, and other metabolites. (a)–(c) Brightfield images for (a) whole droplet dried on aluminum slide (5×); (b) crystalline region (50×); and (c) edge (50×) with acquisition points shown with different symbols: diamonds (on crystal), crosses (off crystal) and triangles (edge). (d) Average spectra from one saliva sample for on crystal (top), off crystal (middle), and edge (bottom) regions respectively with shaded areas representing the interspectral variance within the specimen. Each spectrum is an average of multiple acquisitions obtained with a 785-nm laser using a Renishaw InVia Raman microscope. The scale bar in A is 1 mm in length, whereas the scale bar in B and C are 0.1-mm long.

To confidently assign Raman peaks to donor spectra, we used a saliva model adapted from Sarkar et al.^53^ containing salts, bovine serum albumin, mucin, and other metabolites. The precise composition of the model is listed in Table S2 in the Supplementary Material. We dried the droplet in the same way as the human saliva supernatant samples and observed similar morphology in brightfield images [Figs. 4(a)–4(c)] as in human saliva. Although there are some particles of solid compounds, which had not completely dissolved in the model saliva, the branched crystalline region resembles that of human saliva supernatant. The edge region of model saliva also resembles that of human supernatant in terms of size and the fact that it lacks crystals.

Furthermore, the Raman peaks from the center and the edge of model saliva supernatant [Fig. 4(d)] resembled those of human saliva, suggesting a similar molecular composition. We took spectra of the pure constituents of the model saliva to determine which molecules give rise to peaks in the model saliva and whether these peaks were also present in human saliva supernatant (Figs. S3–S6 in the Supplementary Material). By comparing spectra of human saliva supernatant to model saliva, we could identify Raman peaks due to salts, proteins, and nucleic acids (Table S3 in the Supplementary Material). We found that the spectra from the edge of the drop were primarily composed of peaks due to proteins (phenylalanine at $1003\;{\mathrm{cm}}^{-1}$, amide III at 1200 to $1340\;{\mathrm{cm}}^{-1}$, amide I at 1605 to $1665\;{\mathrm{cm}}^{-1}$). We found that the peak due to nitrate, $1045\;{\mathrm{cm}}^{-1}$, was strong in spectra taken from the central crystalline region of both model saliva and real human saliva supernatant. In the central region compared to the edge, the peak at $1003\;{\mathrm{cm}}^{-1}$ was stronger relative to amide bands, suggesting a contribution to this peak by the salt urea. This is consistent with findings in serum and other biofluids—proteins move toward the edge of the droplet in the drying process while salts may be spread throughout the droplet.^63^^,^^64^

### 3.4 Predictive Modeling for Segmented Saliva Samples

To fully explore the potential of all regions of the dried saliva supernatant matrix for detection of COVID-19, we developed predictive models based on edge, on crystal, and off crystal regions. The large number of COVID-negative saliva samples allowed us to match each COVID-positive sample with a negative sample having approximately the same characteristics in terms of sex at birth, age, COVID symptoms, BMI, and prescription drugs taken (Table 1). This ensured that the clinical characteristics of the samples analyzed were approximately the same between the 33 positive (15 males, 18 females) and 38 negative (20 males, 18 females) samples. Volunteer matching was done to reduce the impact of potential confounding factors. However, there was a slightly higher percentage of analyzed samples from volunteers with comorbidities in the COVID negative group. Comorbidities included optic neuritis, cancer, hypertension, diabetes, anxiety, allergies, and migraines.

ML algorithms were trained using spectra from 33 COVID positive and 38 COVID negative samples. The area of laser excitation was small ($24\;\mu m^{2}$) relative to the area of the droplet (approximately $12\;{\mathrm{mm}}^{2}$). Due to the crystallization process and dilution of biomolecules within saliva, not all spectra necessarily carry molecular information relevant to the COVID status of the patient. As a result, an ML approach needed to be adapted whereby each spectrum acquired from a saliva droplet from a volunteer could be treated independently (Fig. 2). The ML approaches that were employed are based on MIL; specifically, MIL via embedded instance selection (MILES) and MIL with discriminative bag mapping (MILDM). The implementation details of techniques are presented in the Experimental section. For each classification scenario presented below, results are shown using an ROC curve for both MILES and MILDM.

### 3.5 Separating Samples Based on Sex at Birth Increases Accuracy of COVID Detection

**Fig. 5:**
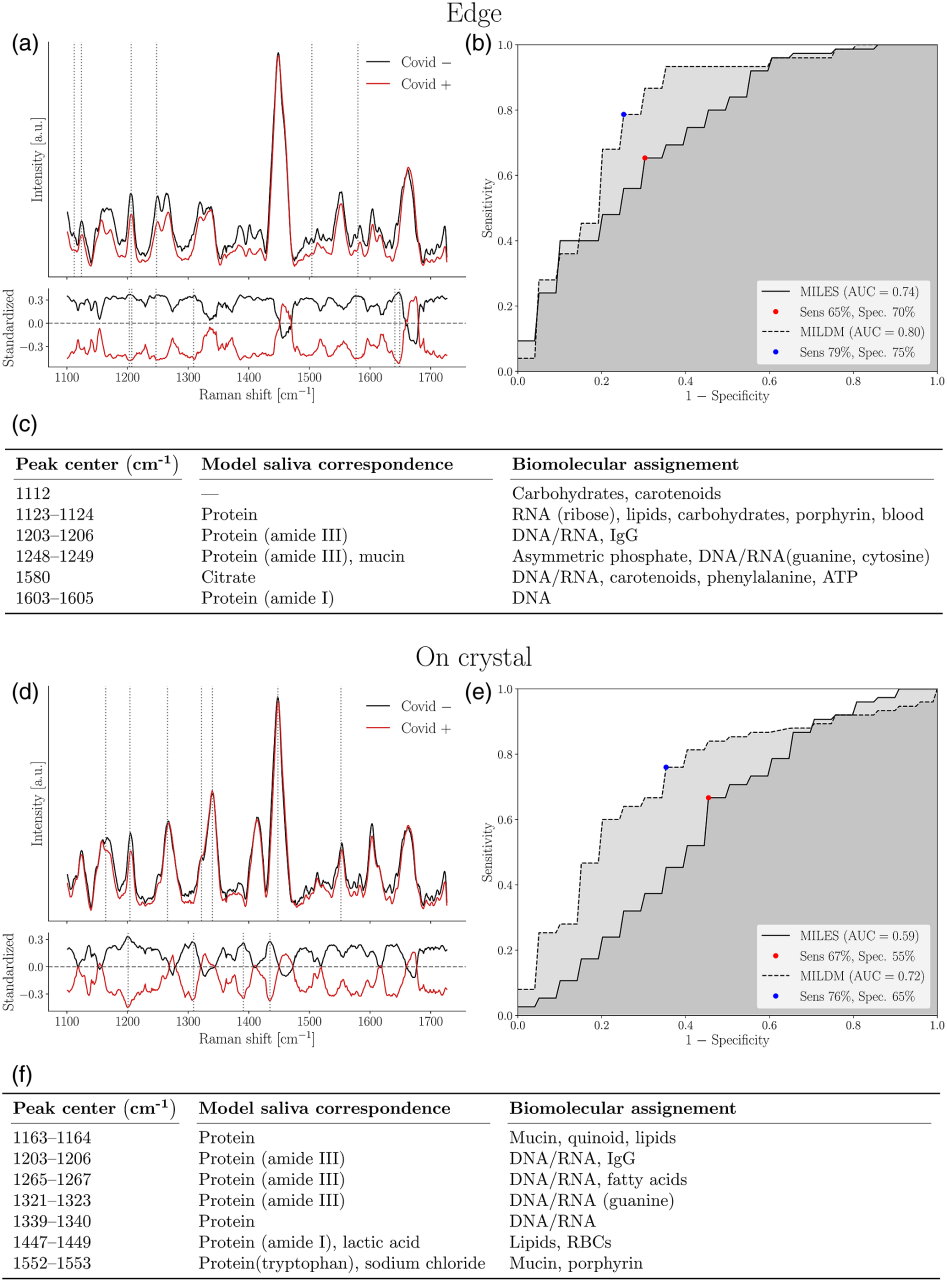
ML model discriminating between COVID-negative and positive saliva supernatant from males using (a)–(c) edge and (d)–(f) on crystal Raman spectra from dried droplets. (a) and (d) Upper frame shows SNV-normalized, background corrected Raman spectra from all volunteers. Main features used in model building designated by dotted lines. Mean spectra from COVID-negative volunteers (n=20, at least eight spectra per volunteer) are shown in black and spectra from COVID-positive volunteers (n=15, at least eight spectra per volunteer) are shown in red. Variance is not shown for reasons of clarity. Bottom frame shows the standardized Raman spectra, where each individual feature has 0 mean and unit variance. (b) and (e) ROC for these models with sensitivity and specificity. (c) and (f) List of features used in model building and their assignments as determined using compounds in model saliva and from literature.

As sex hormones can affect metabolism and immune responses and thus the molecular content of saliva, we separated samples based on sex at birth. In males, we could discriminate between COVID positive and negative saliva supernatant samples with an ROC curve AUC of 0.80 using ML on Raman spectra taken from the edge region [Figs. 5(a) and 5(b)]. This predictive model was built using MILDM (n=35, 15 COVID positive and 20 COVID negative samples), yielding a sensitivity of 79% and a specificity of 75%. Key features used in model building included peaks that can be assigned to carbohydrates, carotenoids, proteins, and nucleic acids [Fig. 5(c)]. In males, models built using the on crystal region had a lower AUC than the edge models (0.72 with MILDM, 15 COVID positive and 20 COVID negative samples) [Figs. 5(d)–5(f)].

**Fig. 6:**
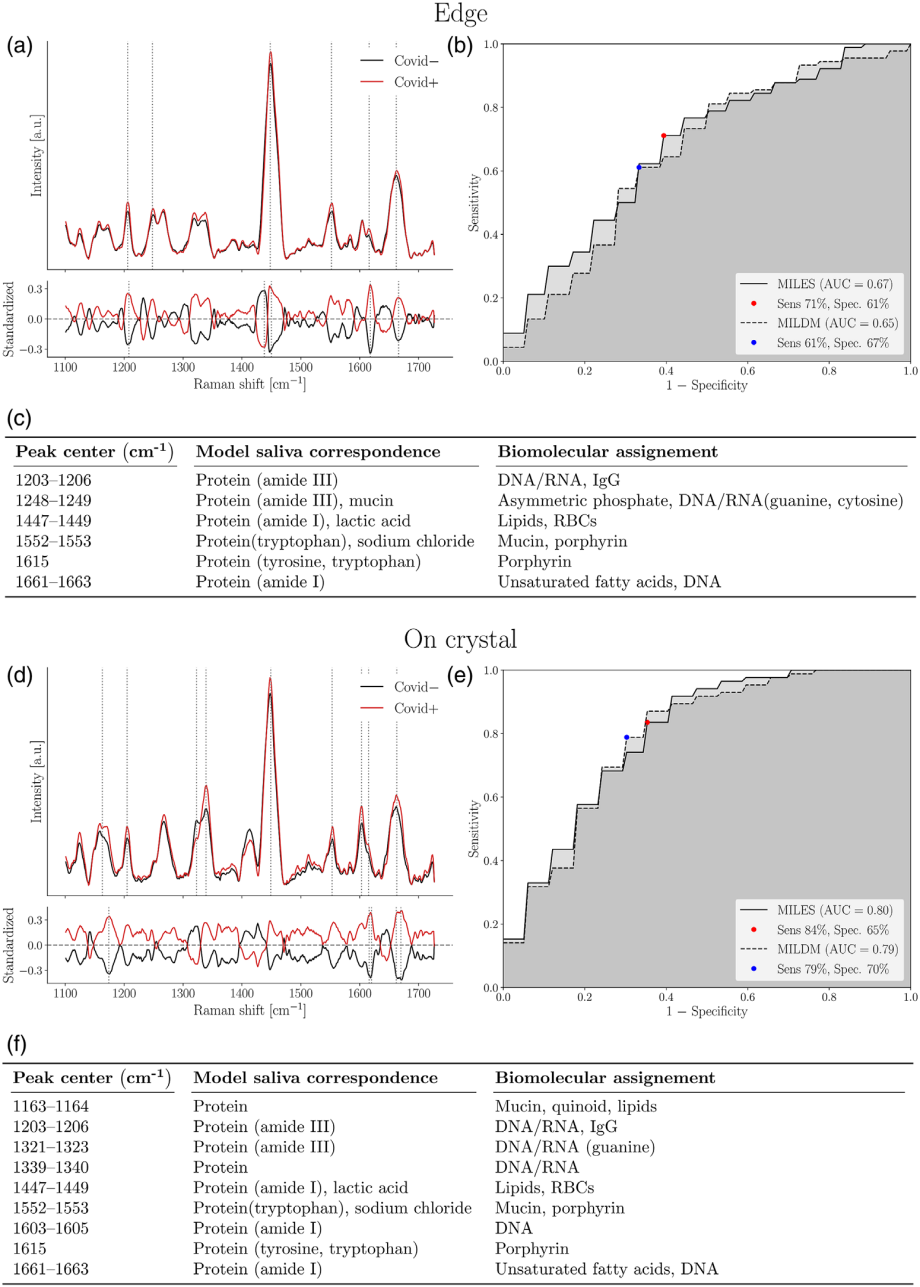
ML model discriminating between COVID-negative and positive saliva supernatant from females using (a)–(c) edge and (d)–(f) on crystal Raman spectra from dried droplets. (a) and (d) Upper frame shows SNV-normalized, background corrected Raman spectra from all volunteers. Main features used in model building designated by dotted lines. Mean spectra from COVID-negative volunteers (n=18, at least nine spectra per volunteer) are shown in black, and spectra from COVID-positive volunteers (n=18 for edge, n=16 for on crystal, at least nine spectra per volunteer) are shown in red. Variance is not shown for reasons of clarity. Bottom frame shows the standardized Raman spectra, where each individual feature has 0 mean and unit variance. (b) and (e) ROC for these models with sensitivity and specificity. (c) and (f) List of features used in model building and their assignments as determined using compounds in model saliva and from literature.

For females, using on crystal spectra, we could discriminate between COVID positive and negative saliva samples with an AUC of 0.80 [Figs. 6(d) and 6(e)]. This model was built using MILES (n=36, 18 COVID-positive and 18 COVID-negative), with a sensitivity of 84% and a specificity of 65%. Key features included peaks that can be assigned to lipids, proteins and nucleic acids [Fig. 6(f)]. In females, models built using the edge region had a lower AUC than the on crystal models (0.67 with MILES, n=36, 16 COVID-positive, 18 COVID-negative) [Figs. 6(a)–6(c)]. This is reflected by the observation that the mean edge spectra of COVID-positive and COVID-negative females have much greater overlap than the mean edge spectra from males, suggesting the molecular composition of the protein-rich edge region of saliva is not altered as severely in females as it is in males.

The best model in males (built using the edge) only shared 1203 to $1206\;{\mathrm{cm}}^{-1}$ and 1603 to $1605\;{\mathrm{cm}}^{-1}$ with the best model in females only. All other features were different.

### 3.6 Discrimination between PCR-Positive and PCR-Negative Samples Regardless of Sex at Birth

**Fig. 7:**
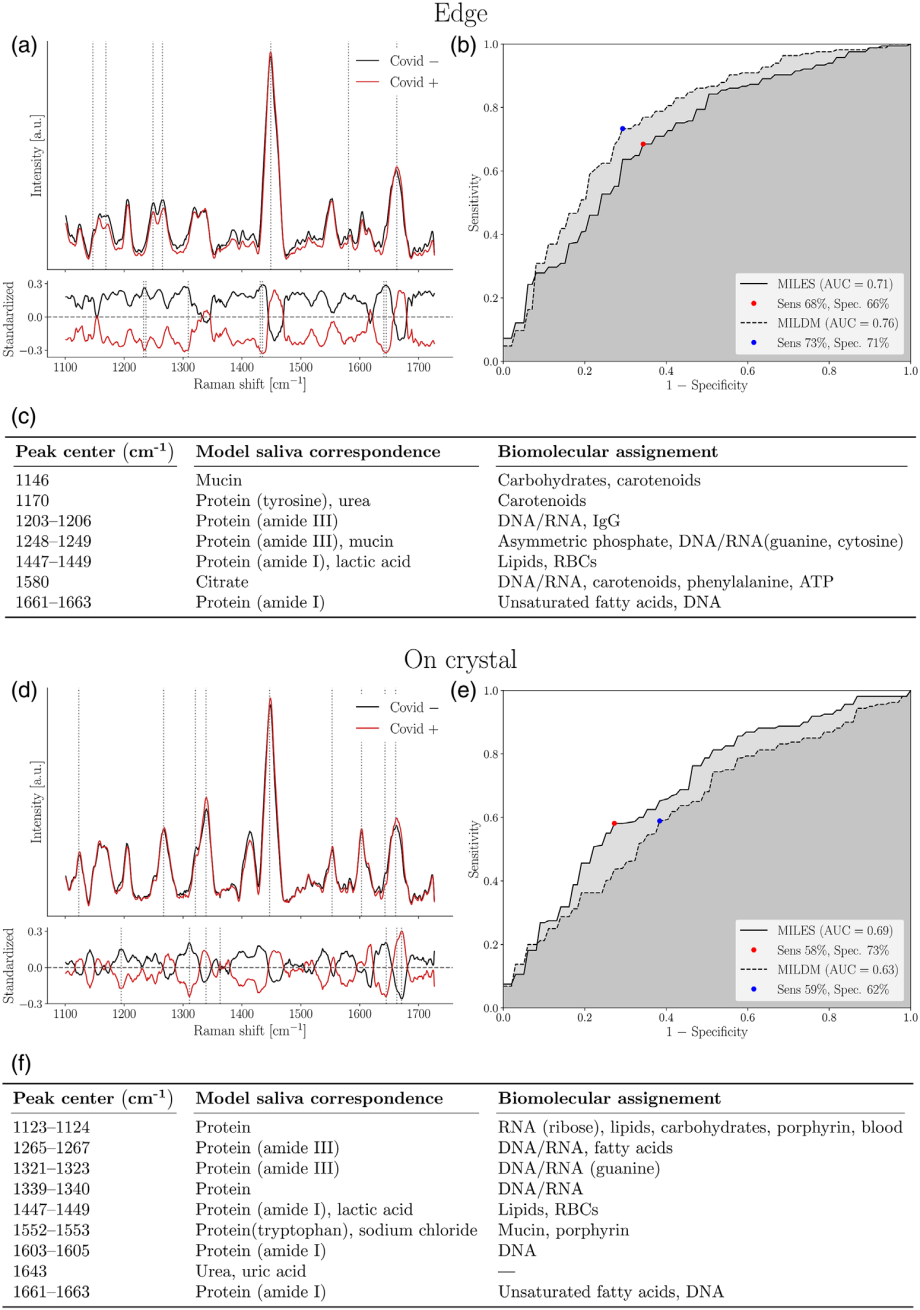
ML model discriminating between COVID-negative and positive volunteer saliva supernatant using (a)–(c) edge and (d)–(f) on crystal Raman spectra from dried droplets. (a) and (d) Upper frame shows SNV-normalized, background corrected Raman spectra from all volunteers. Main features used in model building designated by dotted lines. Mean spectra from COVID-negative volunteers (n=38 for both edge and on crystal, at least nine spectra per volunteer) are shown in black and mean spectra from COVID-positive volunteers spectra (n=33 for edge, 31 for on crystal, at least nine spectra per volunteer) are shown in red. Variance is not shown for reasons of clarity. Bottom frame shows the standardized Raman spectra, where each individual feature has 0 mean and unit variance. (b) and (e) ROC for these models with sensitivity and specificity. (c) and (f) List of features used in model building and their assignments as determined using compounds in model saliva and from literature.

Among all confounding factors including sex at birth, we could discriminate between COVID positive and negative saliva supernatant samples using ML on Raman spectra taken from the edge region with an AUC of 0.76 [Figs. 7(a) and 7(b)]. This predictive model was built using MILDM (n=71, 33 COVID-positive and 38 COVID-negative), yielding a sensitivity of 73% and a specificity of 71%. This reduced the AUC by 0.04 relative to the model built in males alone using the same part of the droplet. Half of the top molecular features are shared between the two models [Figs. 5(c) and 7(c)]. We could discriminate between COVID-19 positive and negative on crystal regions with an AUC of 0.69 using MILES (n=69, 31 COVID-positive, and 38 COVID-negative) [Figs. 7(d) and 7(e)]. This resulted in a reduction in AUC of 0.11 compared to the model built in females only using the same part of the droplet [Fig. 6(e)]. More than 65% of the top features are shared between the two models [Figs. 5(c) and 7(c)]. We could not build a reliable model using spectra from the off crystal region (AUC=0.57). This was also true for models built for males and females considered separately (Figs. S13 and S14 in the Supplementary Material).

Key features used in the edge model for females and males together [Fig. 7(c)] included peaks corresponding to mucin (1146 and 1248 to $1249\;{\mathrm{cm}}^{-1}$), carotenoids (1146, 1170, and $1580\;{\mathrm{cm}}^{-1}$), as well as multiple bands that corresponded to the amide I and III regions in proteins and nucleic acids. In the on crystal region, the top features used to produce the classification model included peaks associated with lipids (1123 to 1124, 1265 to 1267, 1447 to 1449, and 1661 to $1663\;{\mathrm{cm}}^{-1}$), proteins (all peaks except $1643\;{\mathrm{cm}}^{-1}$), nucleic acids, and urea or uric acid ($1643\;{\mathrm{cm}}^{-1}$). One of the key peaks for the on crystal region is the peak at 1339 to $1340\;{\mathrm{cm}}^{-1}$ corresponding to protein and nucleic acids. This is also a peak that is much lower and less distinct in edge regions, suggesting that the regions yield complementary information. Furthermore, only the peaks at 1447 to $1449\;{\mathrm{cm}}^{-1}$ and 1661 to $1663\;{\mathrm{cm}}^{-1}$ were shared features between edge and on crystal predictive models for both sexes together [Figs. 7(c) and 7(f)].

### 3.7 Assessing Effects of Potential Confounding Factors

Building separate models for females and males resulted in higher predictive accuracy than models including both sexes, so we investigated whether we could discriminate between samples based on sex at birth in COVID-negative samples only. The predictive model produced an ROC with an AUC of 0.70 on edge (n=20 males, n=18 females, MILDM) yielding 69% sensitivity, 67% specificity (Figs. S15a–S15c in the Supplementary Material) and 0.80 on crystal yielding 72% sensitivity, 76% specificity (MILES) (Figs. S15d–S15f in the Supplementary Material).

We had also hypothesized that Raman spectroscopy could be used to discriminate between samples from volunteers with respiratory symptoms compared to those without respiratory symptoms, perhaps as the mucus content of saliva could differ between the two. However, we found that we could not build models with high predictive accuracy to discriminate between samples based on whether reported symptoms were considered respiratory symptoms or not (Figs. S16a–S16f in the Supplementary Material). We used samples from all volunteers and grouped non-respiratory symptomatics and asymptomatic samples together into a non-respiratory group and compared the corresponding spectra to those from respiratory symptometics (n=44). Using spectra taken from all volunteers, the AUC was 0.57 in models built using edge spectra (n=44 respiratory, n=23 non-respiratory) and the AUC was 0.56 using MILDM on on crystal spectra (n=43 respiratory, n=23 non-respiratory).

### 3.8 Overfitting Assessment

ML models were compared to models generated using random labels to ensure that models generated using true classification labels were not cases of overfitting. Full results are shown in Tables S4 and S5 in the Supplementary Material and AUC histograms of models generated using random and true classification labels are shown in Figs. S7–S13 in the Supplementary Material. These show that the models produced using the true labels have consistently higher AUCs than those produced using random labels apart from for COVID detection in males using the on crystal region [Fig. S7b in the Supplementary Material, Fig. 5(d)] and the models produced for symptom classification (Fig. S12 and S16 in the Supplementary Material), which have already been discussed.

## 4 Discussion

We were able to discriminate between COVID-positive and COVID-negative saliva samples in a primer-free, label-free way using Raman spectroscopy and ML. This is the first published case to our knowledge of MILES or MILDM applied to Raman spectroscopy. We achieved a sensitivity of 79% and specificity of 75% compared to PCR tests for detecting COVID-19 infection in non-hospitalized males with a range of symptoms, ages, medical conditions, and viral load. We achieved a sensitivity of 84% and specificity of 65% in detecting COVID in non-hospitalized females with similar real-world variables.

Our novel application of MILES and MILDM techniques allowed us to account for possible differences in molecular content between Raman spectra within the same dried droplet. Spatially distinct Raman spectra from a COVID-positive sample may not all contain molecular information associated with COVID positivity and studies have shown that SARS-CoV-2 virions aggregate when dried.^50^ MIL techniques enable assessment of individual spectra rather than assessment of average spectra from each sample, which could result in a loss of information. Application of such a technique could be very useful in Raman microspectroscopic classification. Our proposed rapid screening platform may not rely on microscopy so other ML techniques will be investigated. In a pandemic control context, these ML models could be distributed rapidly and frequently through software updates while development and worldwide distribution of new primers to address variants can take valuable time, which could otherwise be used in limiting the spread of infection.

We found that for males, the best classification model was built using the edge region using features associated with carbohydrates, carotenoids, proteins, and nucleic acids, whereas for females, the best classification model was built using the on crystal region using features associated with lipids, proteins and nucleic acids. The off crystal region—the central region of the saliva droplet outside of the crystals—was not useful, suggesting that compounds associated with COVID-19 were perhaps less concentrated in this region than within the crystals themselves or the edge region.

We have found that the Raman spectrum of the edge region of dried saliva supernatant differs from that of the central region, with the central region containing peaks due to salts and proteins, whereas the edge region is primarily composed of peaks due to proteins (Fig. 3 and Table S3 in the Supplementary Material). This is in agreement with electron microscopy studies of dried solutions of lysozyme and salt mixtures in which lysozyme was shown to accumulate at the edge of dried droplets.^65^ Although many studies claim to use Raman spectroscopy of saliva to diagnose diseases, this is the first published instance to our knowledge in which both edge and center regions (both on crystal and off crystal) have been used in a single study, yielding complementary information in terms of interrogated biomolecular vibrational modes. Moreover, we could not find any other publications in which a saliva model was used to confidently assign molecular features in a Raman spectrum.

The observation that the best model for males is built using different features and spectra from different parts of the droplet compared to females suggests that COVID-19 may elicit different changes in the biomolecular profile of saliva between the sexes. Studies have shown that there are differences in the immune response of males and females to COVID, with females mounting a more robust T cell response, whereas males had higher cytokine levels and are more severely impacted by COVID-19.^66^ Furthermore, ACE2 is expressed in lower levels in liver and lung tissue of women compared to men, which could result in different impacts on metabolism.^67^

We found that sex at birth was a confounding factor in saliva-based COVID-19 detection using label-free Raman spectroscopy as the AUC was reduced in models built from males and females together compared to those built from males and females separately. We could also discriminate between saliva supernatant samples based on sex at birth from COVID-negative females and males with an AUC of 0.80 using the on crystal region. This is consistent with a study by Muro et al.^68^ in which Raman spectroscopy was used to discriminate between 60 samples of whole saliva from males and females. Moreover, the biomolecular composition of saliva has been shown to be different in females and males by NMR spectroscopy.^69^ Levels of glycine, lactic acid, and acetate were all higher in saliva taken from males in that study while with Raman spectroscopy, we observed differences in peaks associated with proteins and nucleic acids, and a peak associated with lactic acid (Fig. 15d–15f in the Supplementary Material).

It seems we cannot discriminate between samples from volunteers with respiratory symptoms compared to volunteers without respiratory symptoms with high sensitivity or specificity using Raman spectroscopy. This may be because the majority of mucus was pelleted along with food debris during the saliva centrifugation process or because mucus content does not differ significantly in saliva supernatant between respiratory and non-respiratory samples. However, as this study occurred during December 2020–February 2021, some volunteers may have exaggerated their symptoms during reporting to obtain a PCR test for non-essential reasons, e.g., travel or meeting family members during the holiday season. Unreliable reporting of symptoms is a key factor to be taken into account during infectious disease research and testing. With symptoms assessed by a trained medical professional and an approximate time since exposure given, we may be able to more accurately determine whether the sensitivity and specificity of our test is affected by whether the volunteer is pre-symptomatic, has respiratory symptoms, non-respiratory symptoms or no symptoms. We also aim to conduct a study in which COVID-tested volunteers could be tested for other respiratory diseases to check the specificity of our test in the face of other respiratory illnesses or simultaneous infections. Another limitation of the study is the mixed PCR tests—saliva and NPG—employed by the COVID testing unit, which was outside of our control. Although previous studies have shown good agreement between the two,^6^ it would be informative to compare saliva PCR, NPG PCR, and Raman saliva tests.

## 5 Conclusions

In conclusion, we have shown that Raman spectroscopy can be used to detect biomolecular changes between COVID-positive and COVID-negative saliva supernatant and that accounting for the sex of the saliva donor can increase the accuracy of predictive models. However, limitations of our Raman microspectroscopy approach include the fact that it was only possible to sample <1% of the full sample area and imaging times were too long for rapid, high throughput COVID screening (17 min per edge region and 1 h for on crystal). It is likely that in this study, we may not have captured the full molecular profile of every saliva drop. Therefore, we have developed in parallel a rapid single-point Raman spectroscopy platform similar to the probe we have already developed and commercialized for detecting brain cancer^25^^,^^70^^,^^71^ for use in future studies for biofluids. This can image a whole droplet within a few seconds and is portable, affordable, and suitable for high throughput on-site screening. PCR tests typically take at least 2.5 h from sample collection to result.^10^ As sensitivity and specificity of ML models can be tweaked along the ROC curve, sensitivity could be traded for slight losses in specificity in a Raman-based screening test. In a pandemic control context, a high sensitivity screening technique would be desirable, as potential positives could have a follow-up PCR test to confirm positivity. Our rapid screening technique could be followed by a more specific PCR test or be employed as a reagent-free alternative to lateral flow tests, depending on government policy.

The platform could also be used for detection of other infectious diseases or COVID variants simply by retraining the ML algorithms on new samples. This would also enable us to test the generalizability of our methodology on multiple spectrometers.

Saliva is a complex medium and multiple factors can affect the Raman spectrum. However, even while taking into account sex at birth, the maximum AUC in our study was 0.80. This suggests that there are further confounding factors. We are currently expanding our analysis to image the remaining 475 COVID-negative samples to take such factors into account using the single point platform. We will be thoroughly assessing the effects of more confounding factors such as age, diet, smoking, and comorbidities on the salivary Raman fingerprint and evaluating whether these variables can impact the accuracy of detecting COVID-19. In the future, with our more rapid device, we aim to carry out further testing using independent test sets from different testing centers, allowing us to gather more spectral data and associated information about confounding variables. This will enable us to look into factors that could affect hormone levels such as pregnancy, medical conditions, prescription medications and surgeries, and assess their impact on the Raman spectrum of saliva.

In summary, our volunteer-matched study demonstrates that there are molecular differences between saliva of COVID-positive and COVID-negative individuals that may be detected using Raman microspectroscopy amongst confounding factors. We next aim to create a single point Raman spectroscopy platform that could be easily integrated into the viral screening workflow.

## Code, Data and Materials Availability

Anonymized Raman spectral files are available upon request.
