# Supplementary material for: Saliva-based detection of COVID-19 infection in a real-world setting using reagent-free Raman spectroscopy and machine learning
Source: J Biomed Opt. 2022 Feb 9;27(2):025002. doi: 10.1117/1.JBO.27.2.025002 (PMC8825664; doi:10.1117/1.JBO.27.2.025002)
Supplement: Supplementary file 1 [file JBO_027_025002_SD001.pdf]

Supplementary Materials for

Saliva-based detection of COVID-19 infection in a real-world setting using reagent-free Raman spectroscopy and machine learning

Katherine J. I. Ember†, François Daoust‡, Myriam Mahfoud‡, Frédérick Dallaire, Esmat Zamani, Trang Tran, Arthur Plante Mame-Kany Diop, Tien Nguyen, Amelie St-Georges-Robillard, Nassim Ksantini, Julie L. Lanthier, Antoine Filiatrault, Guillaume Sheehy, Dominique Trudel, Frédéric Leblond*

*Corresponding author. Email: frederic.leblond@polymtl.ca

† First author
‡Equally contributing authors

This PDF file includes:

Figs. S1 to S8
Tables S1 to S4

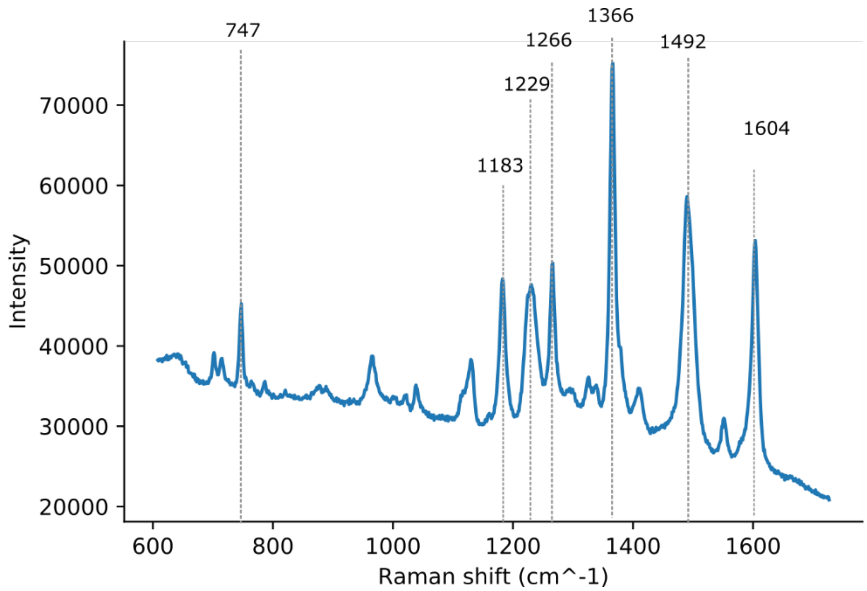

Fig. S1.

A Raman spectrum from lipstick contamination in a saliva supernatant sample from a COVID-19 negative volunteer. Although the saliva donor was wearing a red lipstick from Max factor, the Raman spectrum is remarkably similar to that of red Bourjois 15 (pure and not in saliva) which has peaks at or one wavenumber away from 747, 1183, 1229, 1266, 1366, 1492 and 1604 cm^{-1} [Ref. 72]

Table S1.

Available viral load data of COVID positive samples for all samples for which we were able to obtain viral load data from the testing centre

Sample	Ct Target 1	Ct Target 2
1	18.2	18.2
2	30.6	30.6
3	19.2	19.4
4	18	18
5	27.1	27.1
6	17.9	17.6
7	33.5	35.6
8	20.2	20.2
9	21.7	22.4
10	20.2	20.3
11	24.1	23.8
12	15.5	15.5
13	32.7	34
14	0	35.3
15	33.8	35.4
16	0	36.3
17	32.6	34.5
18	20.1	20.5

Table S2.
Concentrations of compounds added to distilled H_2O to produce model saliva.

**Compound**	**Concentration (g/L)**	**Supplier (product code)**
Sodium chloride	1.594	Fisher Scientific (BP358-1)
Ammonium nitrate	0.057	Cedarlane (A300-500G)
Potassium phosphate	0.636	Fisher Scientific (P382-500)
Potassium chloride	0.202	Sigma Aldrich (P9451-500G)
Potassium citrate	0.308	Sigma Aldrich (60153-1KG-F)
Uric acid sodium salt	0.021	Sigma Aldrich (U-0881)
Urea	0.198	VWR Life Science (VWR0378)
Lactic acid sodium salt	0.146	J&K Scientific (148977)
Glucose	0.014	Sigma (G8270-1KG)
Human serum albumin	4	Cedarlane (CLPRO534-2)
Bovine submaxillary mucin	1	Sigma (A2153)

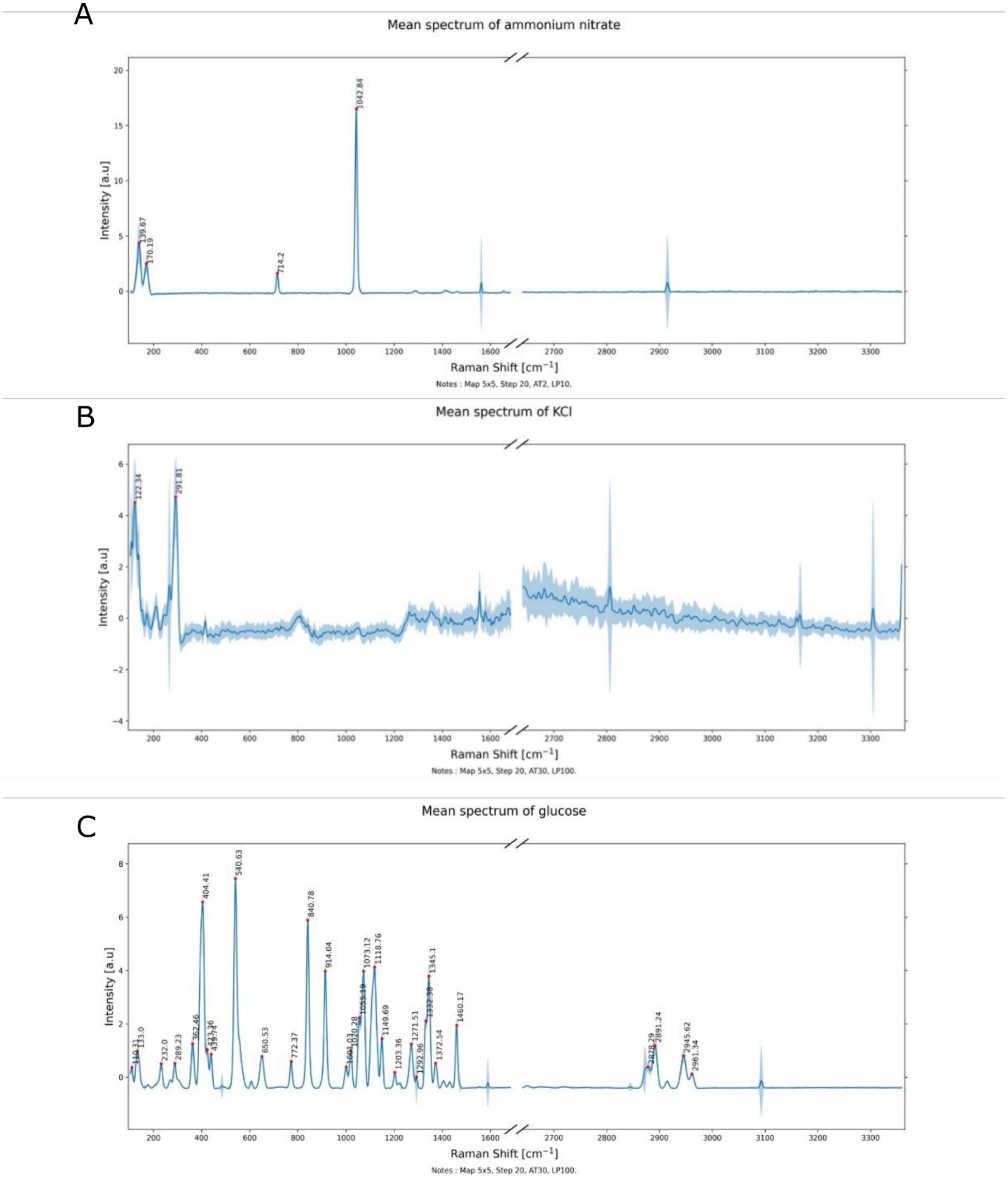

Fig. S3.
Raman spectra from a) ammonium nitrate, b) potassium chloride and c) glucose. Spectra were taken using 785 nm excitation with a Renishaw InVia Raman spectrometer from solid compounds placed on an aluminum slide.

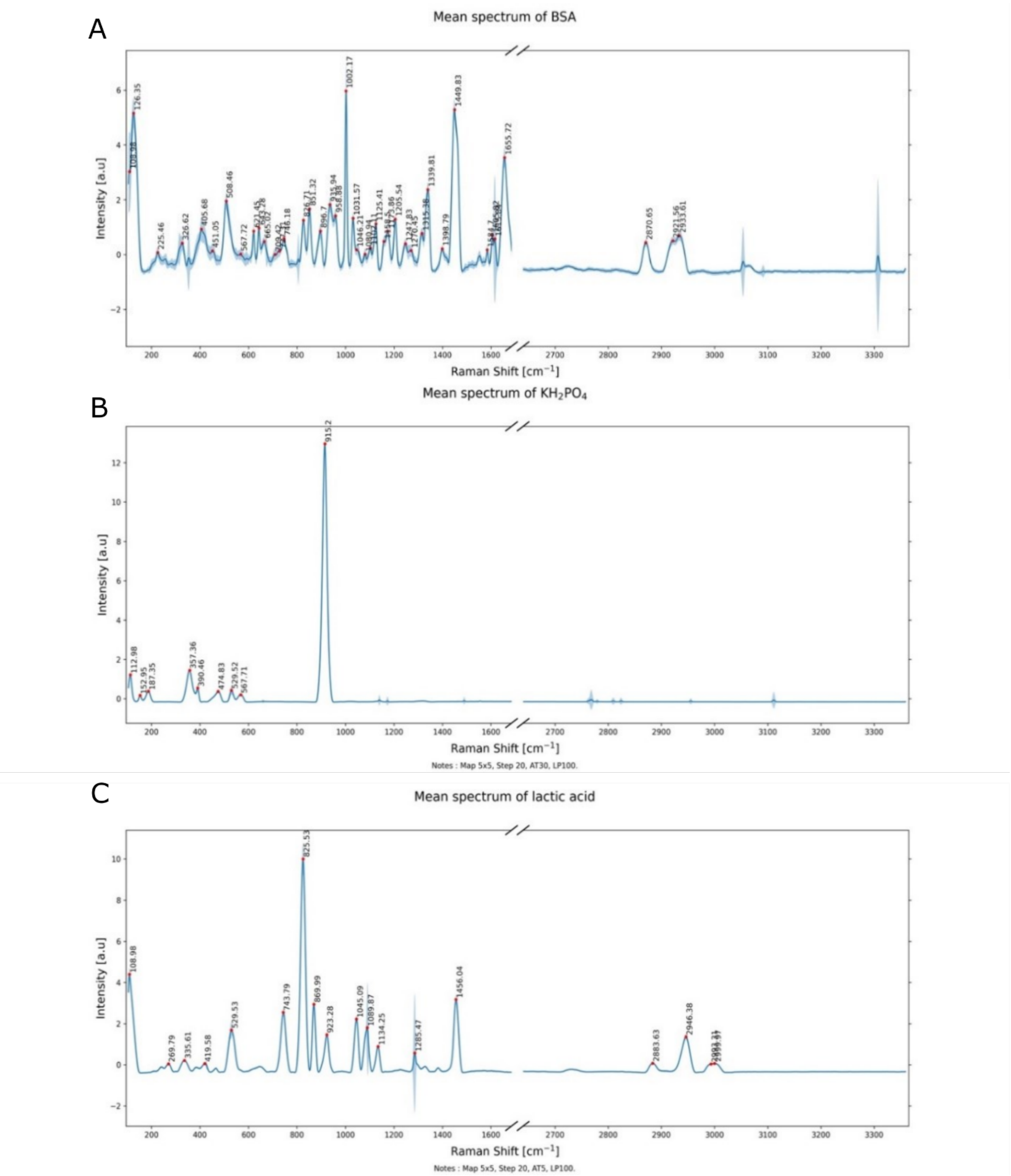

Fig. S4.

Raman spectra from a) bovine serum albumin, b) potassium phosphate and c) lactic acid. Spectra were taken using 785 nm excitation with a Renishaw InVia Raman spectrometer from solid compounds placed on an aluminum slide.

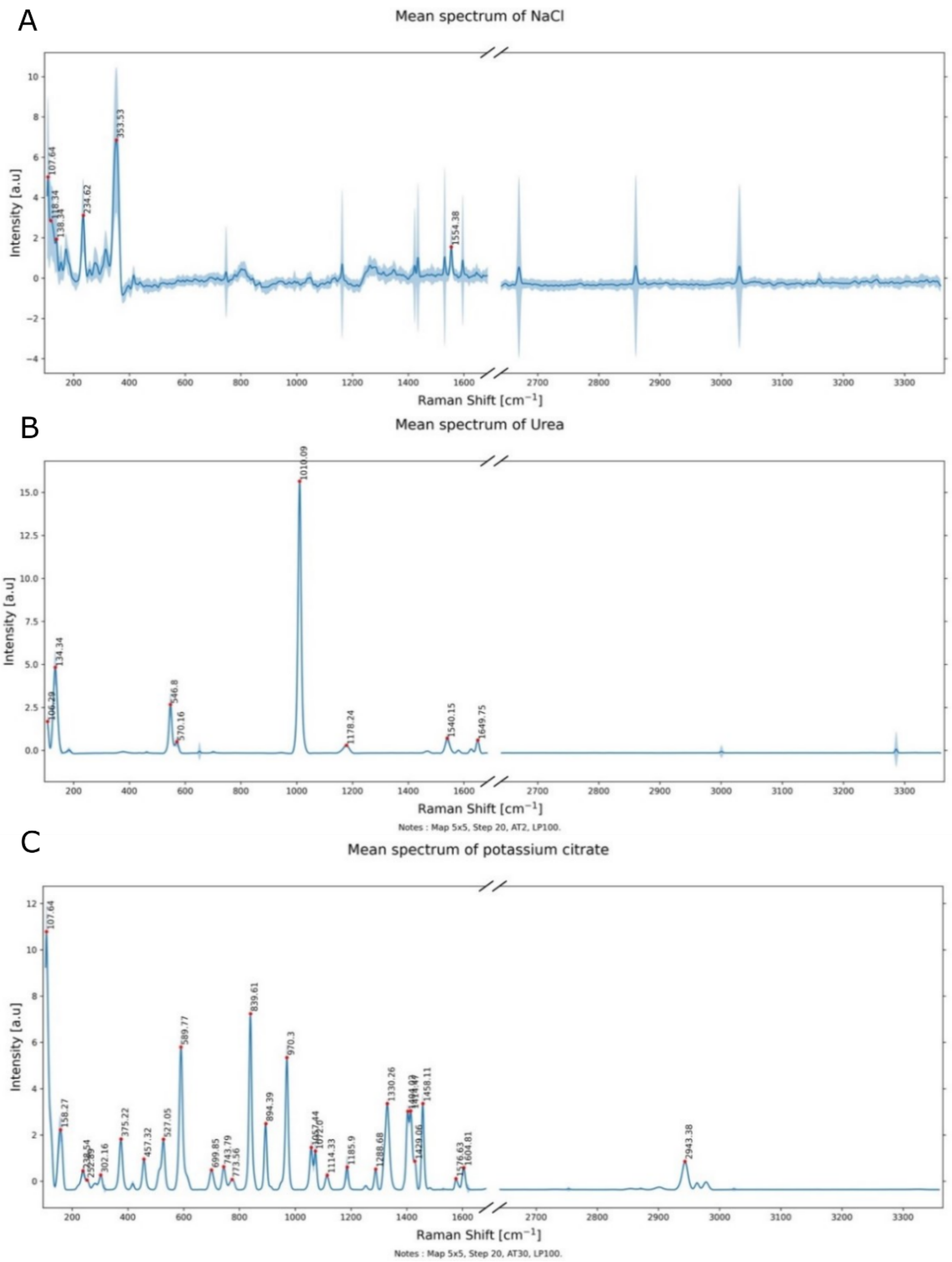

Fig. S5.

Raman spectra from a) sodium chloride, b) urea and c) potassium citrate. Spectra were taken using 785 nm excitation with a Renishaw InVia Raman spectrometer from solid compounds placed on an aluminum.

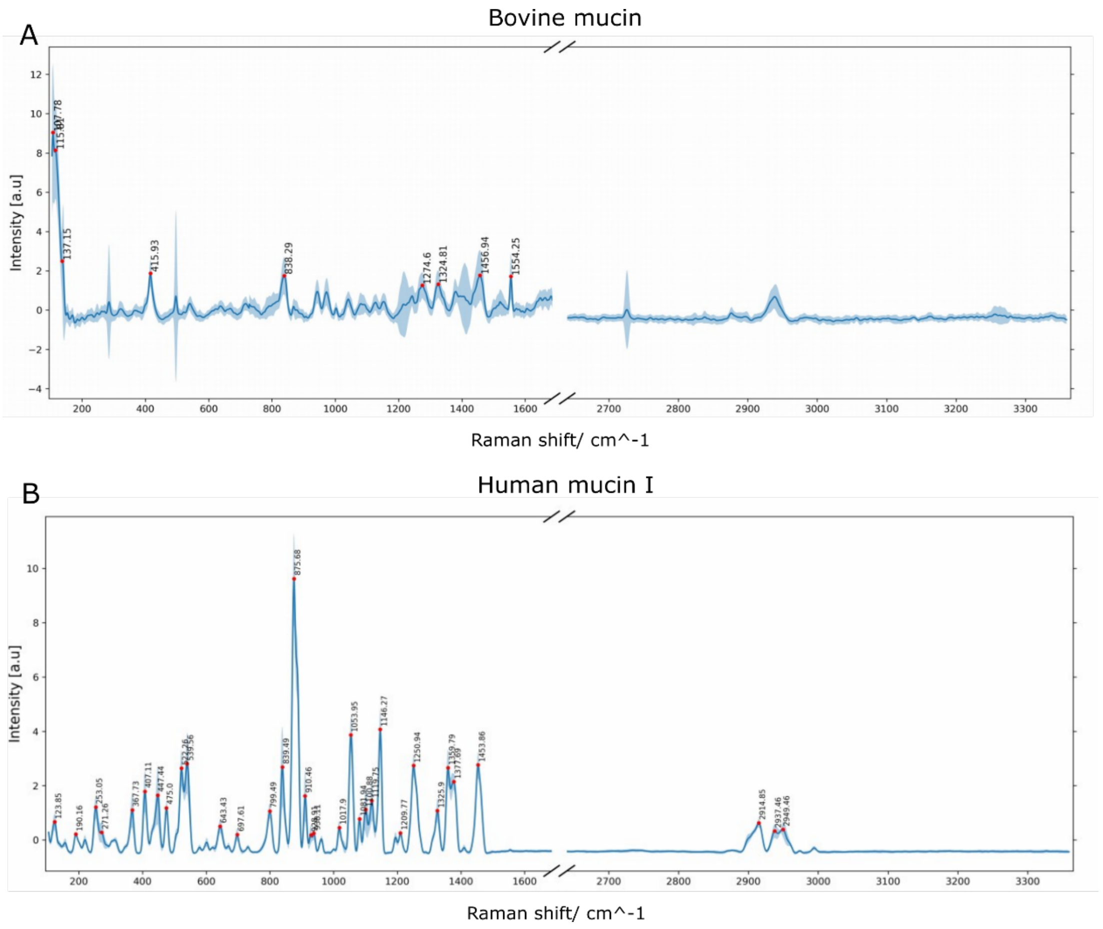

Fig. S6.
Raman spectra from a) bovine submaxillary mucin and b) human mucin I. Spectra were taken using 785 nm excitation with a Renishaw InVia Raman spectrometer from solid compounds placed on an aluminum slide.

Table S3.
Table of the Raman peaks present in Raman spectra from a dried droplets of model saliva and human saliva supernatant (shown in Figs 2 and 3 of main paper) in addition to their location (presence in edge or center of the droplets) and the assignments from model saliva constituents. “Center” refers to on crystal and off crystal regions as peaks were present in both. Additional possible contributions to bands from literature are taken from Refs. 35, 62, 73, and 74. Raman peaks for some molecules may be undetectable as the peaks are either not present with sufficient signal to noise ratio, or because the peaks lie under the shoulders of broad adjacent peaks.

**Raman peak / cm^{-1}**	**Location of peak in model saliva**	**Location of peak in human saliva supernatant**	**Biomolecular assignments**	
			**Band assignment based in peak position from consituents of model saliva**	**Additional possible contributions to bands from literature**
623	Edge	Edge, Center	Protein (phenylalanine) or uric acid	
646	Edge	Edge, Center	Protein (tyrosine, phenylalanine), glucose	
718	Undetected	Edge, center		DNA, phospholipids
731	Undetected	Edge		Adenine, phosphatidylserine
746	Edge	Center	Protein	DNA
760	Undetected	Edge, center		Tryptophan, ethanolamine
790	Center	Edge, center	Glucose, uric acid	Phosphodiester
805	Undetected	Edge, center		Uracil
827	Undetected	Edge, center	Protein	Phosphodiester in DNA
835	Edge	Undetected		Amine
853	Edge, center	Edge, center	Glucose, potassium citrate	Tyrosine, proline, polysaccharides
876	Undetected	Edge, center	Phosphate, human mucin I	Choline, phospholipids
890	Undetected	Center		Proteins, carbohydrates
900	Edge	Undetected		Carbohydrates

924	Undetected	Edge, center	Phosphate, glucose and protein (proline), lactic acid	
935	Center	Edge	Protein (proline, valine)	Glycogen
942	Edge	Undetected		Carbohydrates
957	Edge	Center	Protein	Hydroxyapetite, carotenoid, cholesterol
1003	Center (broader) and edge (narrower)	Center and edge	Phenylalanine/protein, urea	NADH
1031	Center and edge	Edge	Protein (phenylalanine)	Phospholipids
1045	Edge (weak), center	Edge (weak), center (strong)	Nitrate and protein (phenylalanine), uric acid, lactic acid, human mucin I	Phosphate, carbohydrate
1082	Edge, center (weak)	Center (weak)	Protein, glucose	Carbohydrates, nucleic acids, phospholipids, ATP
1101	Edge	Edge	Protein	C-N, lipids
1112	Undetected	Edge		Carbohydrates, carotenoids
1125	Edge, center (weak)	Edge, center	Protein	Lipid, RNA (ribose), carbohydrate, blood, porphyrin
1146	Edge	Edge, center	Human mucin I	Carbohydrates, carotenoids
1156	Edge	Edge, center	Protein	Carotenoids
1173	Edge, center (weak)	Edge	Protein (tyrosine), urea	Carotenoids
1203	Center (weak)	Center (weak)		Nucleic acids, amide III
1206	Edge	Edge	Protein (amide III)	Nucleic acids, IgG
1250	Undetected	Edge	Protein (amide III), human mucin I	Asymmetric phosphate, DNA/ RNA (guanine, cytosine)
1266	Undetected	Edge, center	Protein (amide III)	Nucleic acids, fatty acids

1319	Edge	Edge, center	Protein (amide III)	Nucleic acids (guanine)
1338	Edge and center	Edge, center	Protein (amide III)	Nucleic acids
1401	Center	Edge		Methyl groups, aspartate, glutamate
1417	Center	Edge (weaker), center (stronger)		Aspartate, glutamate
1449	Edge (strong), center	Edge and center	Protein (amide I), lactic acid	Lipids, red blood cells, aromatic carbonds
1512	Undetected	Edge, center		DNA, cytosine
1519	Undetected	Edge		Carotenoid, porphyrin
1553	Center (strong relative to edge), edge (v weak)	Edge, center	Protein (tryptophan, amide II), sodium chloride	Mucin, porphyrin
1574		Edge		DNA
1584	Edge	Edge	Citrate	Phenylalanine, ATP, carotenoids, DNA/RNA
1600	Center	Undetected		Amide I, phenylalanine
1605	Edge	Edge, center	Protein (amide I)	DNA
1616	Edge	Edge, center (in shoulder of peak)	Protein (tyrosine, tryptophan)	Porphyrin
1655	Edge (strong), center (v weak)	Undetected	Protein (amide I), urea, uric acid	Lipid
1665	Undetected	Edge, center	Protein (amide I)	Unsaturated fatty acids, DNA

Table S4.
Characteristics of spectra used in generation of predictive models in this study. “Spectra type” refers to whether all or a cropped region of the spectra was used in the model. “Crop” refers to spectra that have had the region with high variance before 1100 cm^{-1} removed. “Region” refers to which region of the dried droplet the spectra were acquired from. In the column for sex, “M” refers to males and “F” refers to females. “People” refer to number of volunteers and hence the number of saliva samples involved in each model.

Spectra type	Model no.	Region	Sex	Classification label	COVID status	No. of people	No. of spectra	AUC MILES	AUC MILDM
Crop	1	Edge	M	Covid	All	35	342	0.74	0.80
Crop	2	On crystal	M	Covid	All	35	347	0.59	0.72
Crop	N/A	Off crystal	M	Covid	All	35	332	0.57	0.62
Crop	3	Edge	F	Covid	All	36	320	0.67	0.65
Crop	4	On crystal	F	Covid	All	34	300	0.80	0.79
Crop	N/A	Off crystal	F	Covid	All	35	332	0.58	0.57
All	5	Edge	Both	Covid	All	71	702	0.68	0.67
Crop	6	Edge	Both	Covid	All	71	702	0.71	0.76
Crop	7	On crystal	Both	Covid	All	69	687	0.69	0.63
Crop	8	Off crystal	Both	Covid	All	69	668	0.59	0.57
Crop	9	Edge	Both	Sex	Negative	38	702	0.69	0.70
Crop	10	On crystal	Both	Sex	Negative	37	373	0.80	0.67
Crop	N/A	Off crystal	Both	Sex	Negative	37	369	0.61	0.59
Crop	11	Edge	Both	Symptoms	All	71	702	0.53	0.57
Crop	12	On crystal	Both	Symptoms	All	69	687	0.55	0.56
Crop	N/A	Off crystal	Both	Symptoms	All	69	668	0.54	0.56

Table S5.
Area under curve (AUC) values for receiver operating characteristic (ROC) curves generated using both MILES and MILDM in the study, produced for predictive models listed in Table S4. Mean AUC with the true label differs from the AUC given in Table S4 because AUCs in S4 are using optimized hyperparameters .

Model number	Mean AUC MILES true labels	Mean AUC MILES random labels	Mean AUC MILDM true labels	Mean AUC MILDM random labels	Figure in main text
1	0.658	0.494	0.680	0.498	Fig 5A-C
2	0.498	0.488	0.463	0.489	Fig 5D-F
3	0.562	0.476	0.541	0.481	Fig 6A-C
4	0.649	0.485	0.626	0.487	Fig 6D-F
5	0.606	0.488	0.531	0.491	N/A
6	0.625	0.493	0.605	0.490	Fig 7A-C
7	0.558	0.488	0.494	0.490	Fig 7D-F
8	0.552	0.490	0.494	0.494	N/A
9	0.606	0.491	0.611	0.490	Fig S15 A-C
10	0.620	0.489	0.547	0.488	Fig S15 D-F
11	0.461	0.487	0.476	0.491	Fig S16 A-B
12	0.460	0.488	0.466	0.486	Fig S16 C-D

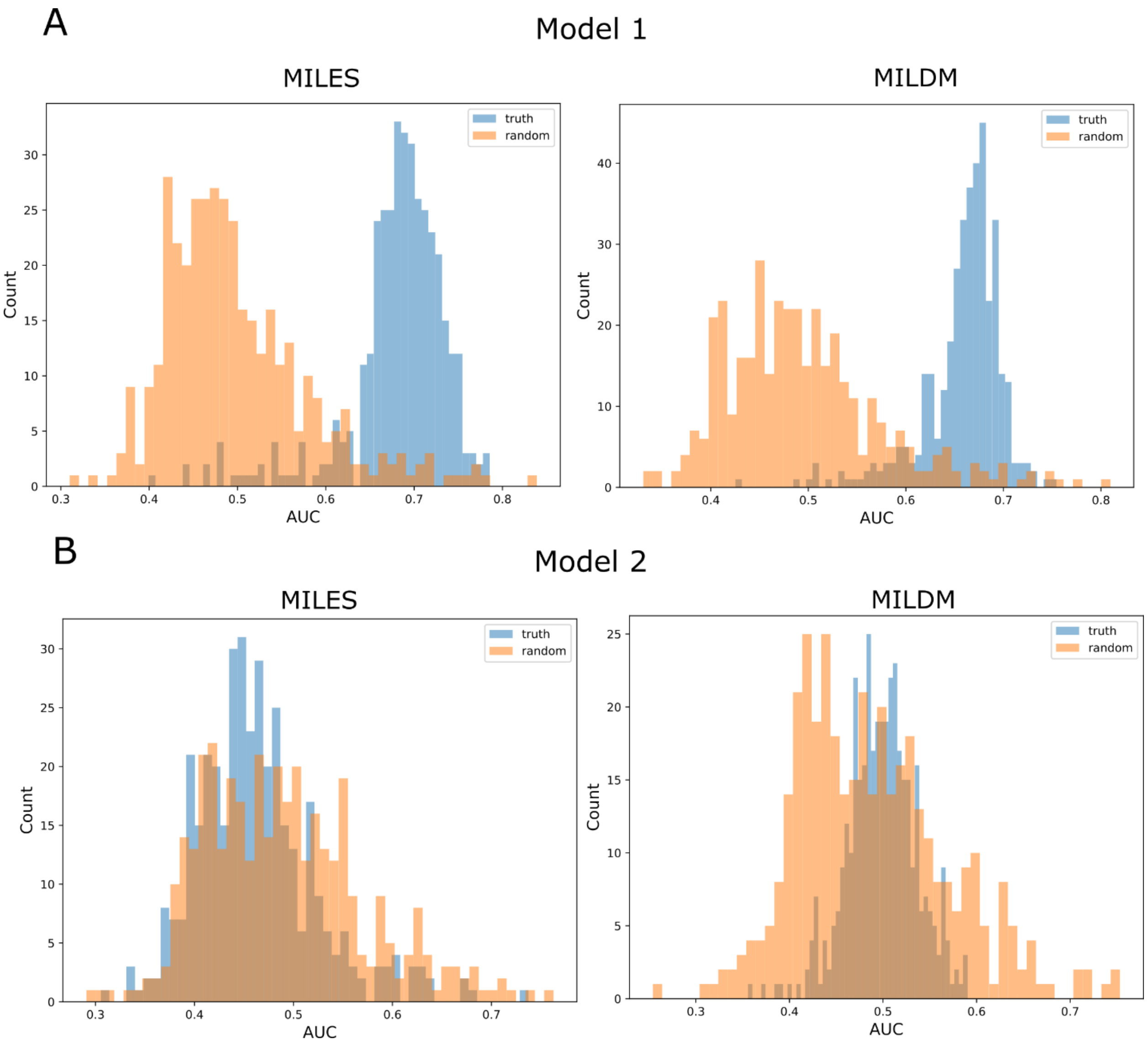

Fig. S7. Histograms plotting each area under curve (AUC) calculated from receiver operating characteristic (ROC) curves for classification models with random and true data labels. 96 classification models were run using random labels (orange bars) compared to true labels (blue bars). These models discriminate between Raman spectra from dried saliva droplets from males based on COVID status using spectra taken between 1100 and 1726 cm^{-1}. (A) Model 1 used spectra taken from the “edge” region of a dried droplet, (B) Model 2 used spectra from the “on crystal” region. Figures on the left side show results using multiple instance learning (MILES) and figures on the right side show results using multiple instance learning with discriminative mapping (MILDM).

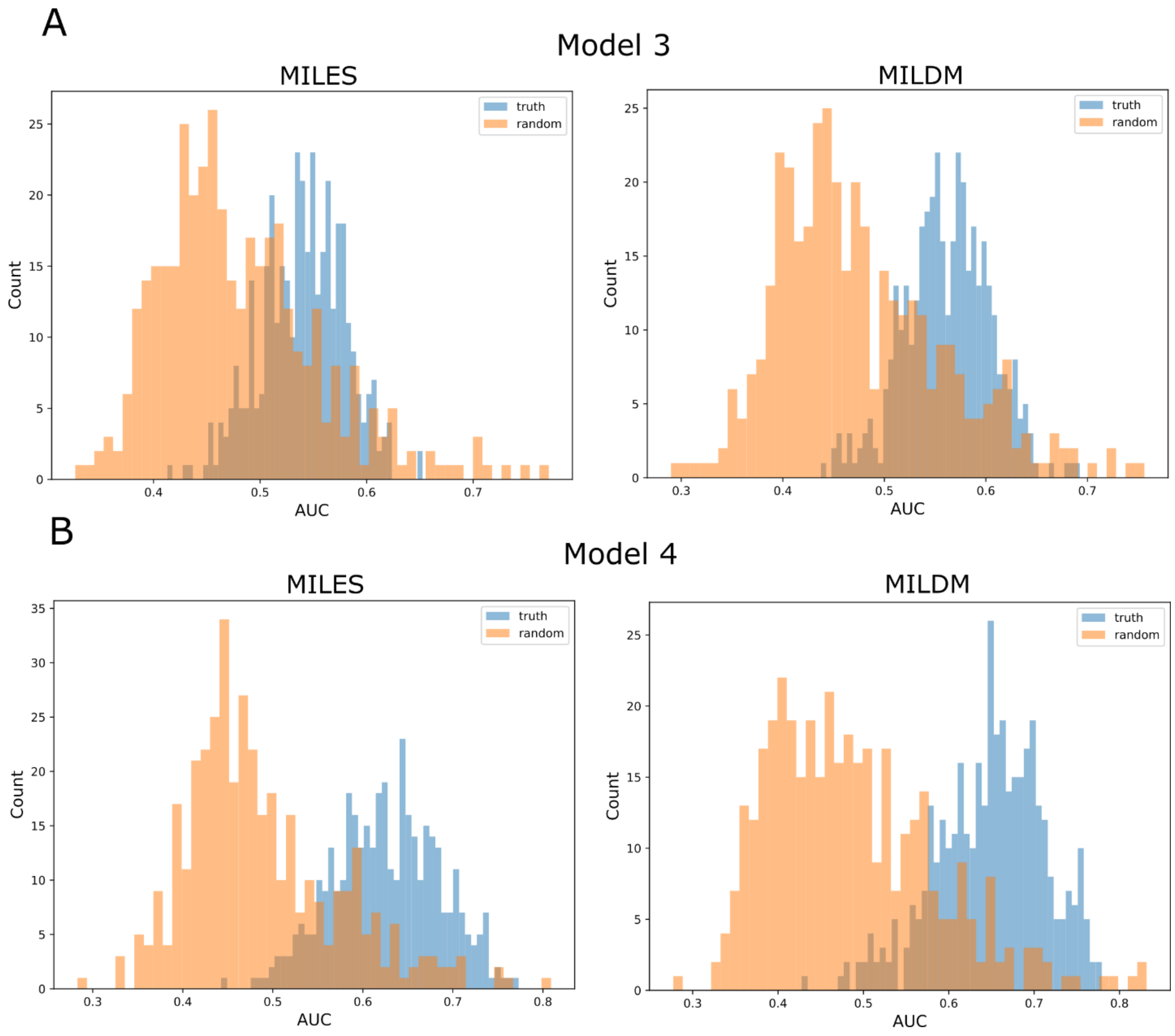

Fig. S8. Histograms plotting each area under curve (AUC) calculated from receiver operating characteristic (ROC) curves for classification models with random and true data labels. 96 classification models were run using random labels (orange bars) compared to true labels (blue bars). These models discriminate between Raman spectra from dried saliva droplets from males based on COVID status using spectra taken between 1100 and 1726 cm^{-1}. (A) Model 3 used spectra taken from the "edge" region of a dried droplet, (B) Model 4 used spectra from the "on crystal" region**.** Figures on the left side show results using multiple instance learning (MILES) and figures on the right side show results using multiple instance learning with discriminative mapping (MILDM).

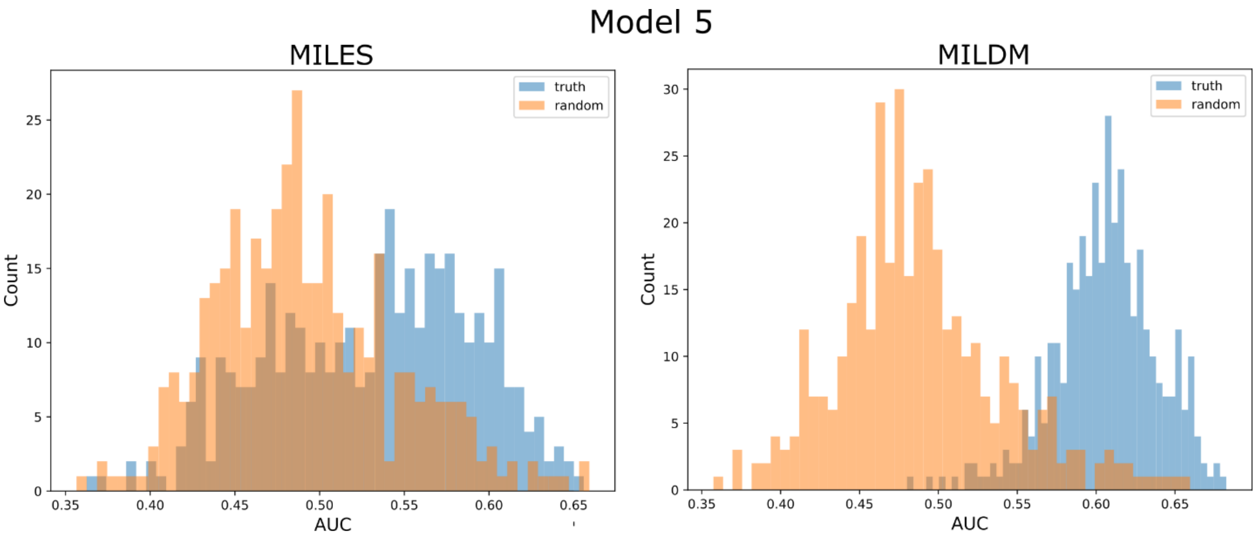

Fig. S9. Histogram plotting each area under curve (AUC) calculated from receiver operating characteristic (ROC) curves for classification models with random and true data labels. 96 classification models were run using random labels (orange bars) compared to true labels (blue bars). Model 5 discriminated between Raman spectra from saliva samples based on COVID status using spectra from the “edge” region for both sexes taken between 602 and 1726 cm^{-1}. Figure on the left side shows results using multiple instance learning (MILES) and figure on the right side shows results using multiple instance learning with discriminative mapping (MILDM).

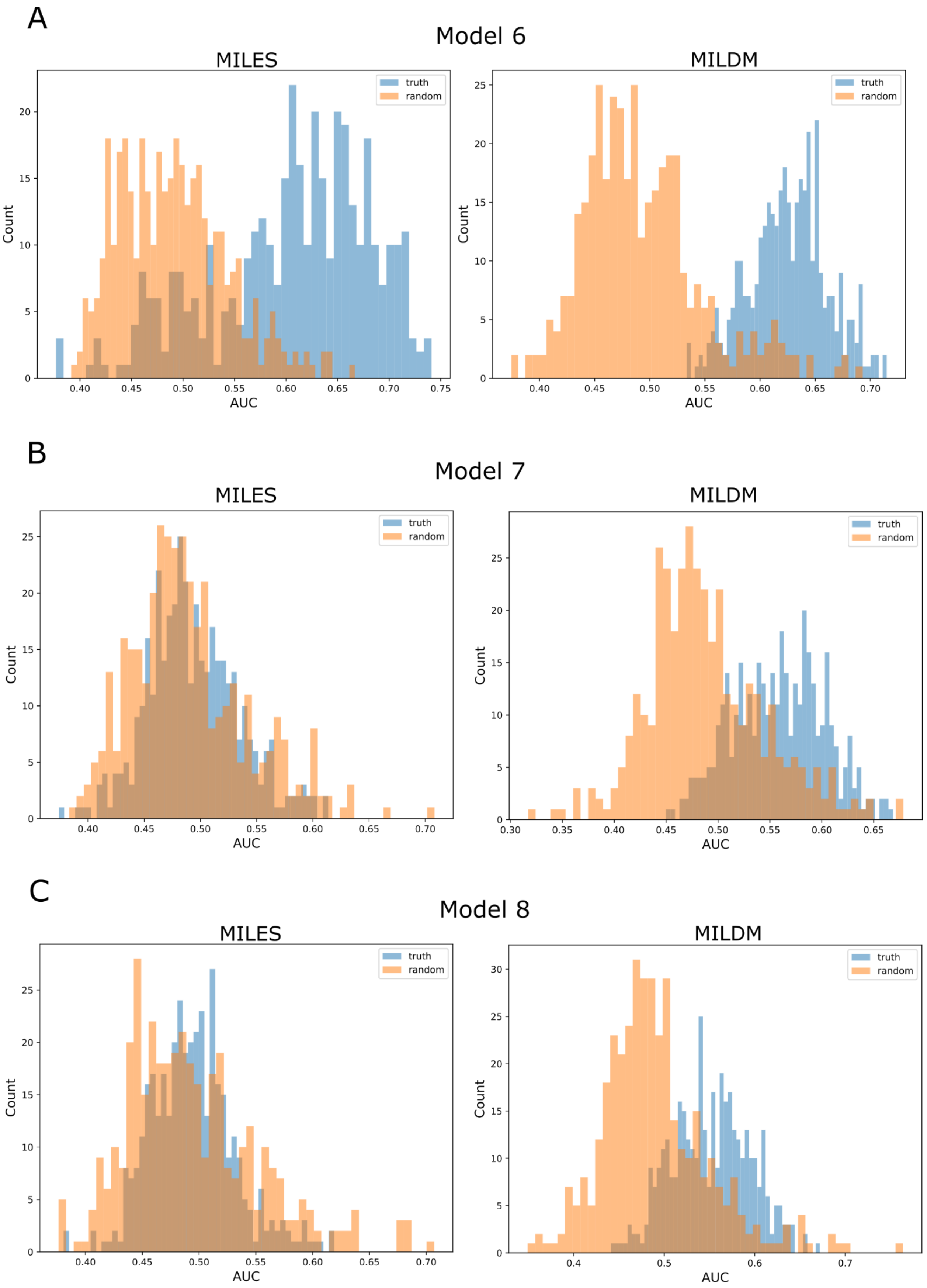
A
Model 6
MILES
MILDM
B
Model 7
MILES
MILDM
C
Model 8
MILES
MILDM
truth
random
Count
AUC

Fig. S10. Histograms plotting each area under curve (AUC) calculated from receiver operating characteristic (ROC) curves for classification models with random and true data labels. 96 classification models were run using random labels (orange bars) compared to true labels (blue bars). These models discriminate between Raman spectra from dried saliva droplets from both sexes based on COVID status using spectra taken between 1100 and 1726 cm^{-1}. (A) Model 6 used spectra taken from the "edge" region, (B) Model 7 used spectra from the "on crystal" region and (C) Model 8 used spectra from the "off crystal" region. Figures on the left side show results using multiple instance learning (MILES) and figures on the right side show results using multiple instance learning with discriminative mapping (MILDM).

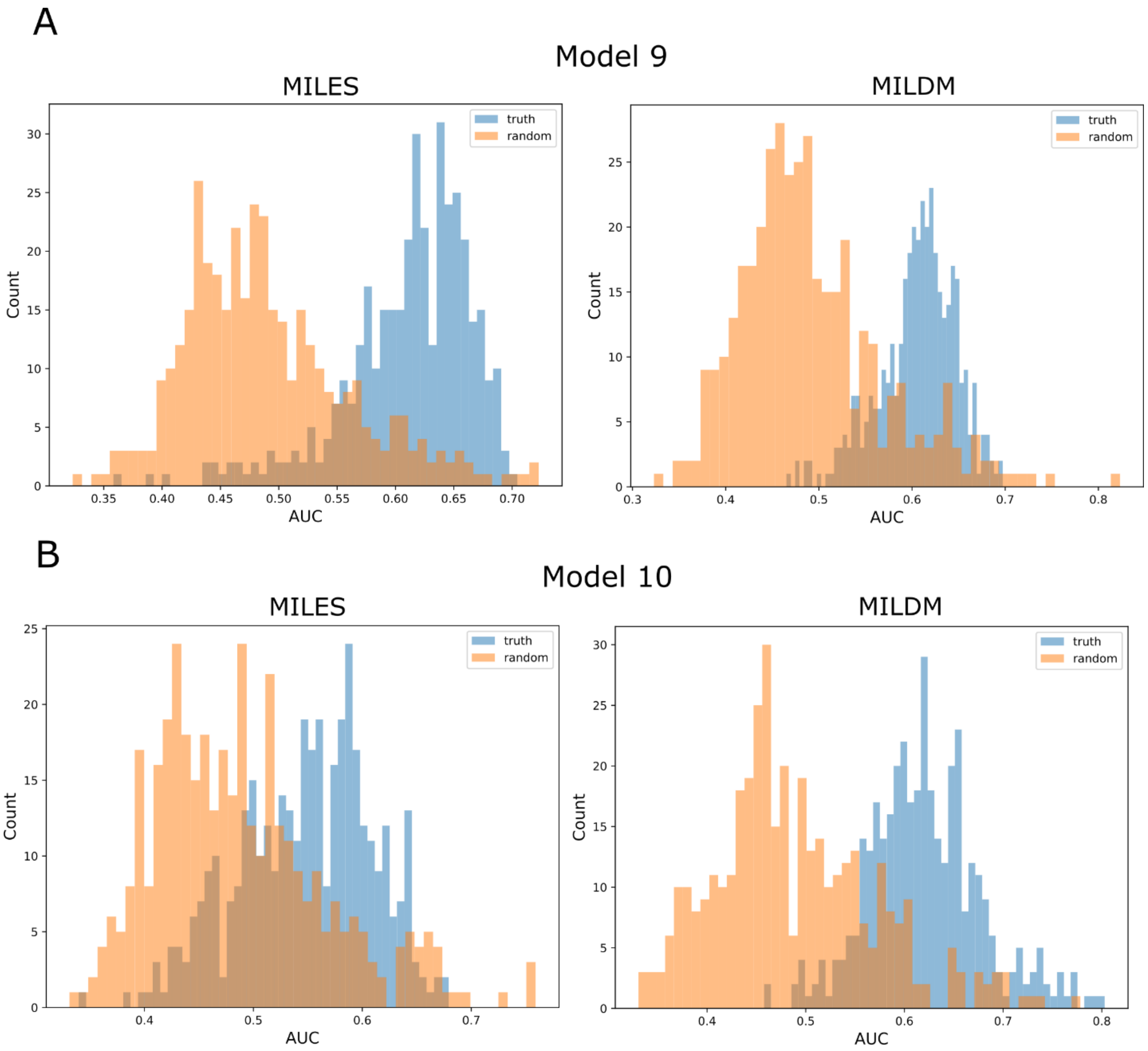

Fig. S11. Histograms plotting each area under curve (AUC) calculated from receiver operating characteristic (ROC) curves for classification models with random and true data labels. 96 classification models were run using random labels (orange bars) compared to true labels (blue

bars). These models discriminate between Raman spectra from dried saliva droplets from COVID-negative volunteers based on sex at birth taken between 1100 and 1726 cm^{-1}. (A) Model 9 used spectra taken from the “edge” region of a dried droplet from both sexes, (B) Model 10 used spectra from the “on crystal” region. Figures on the left side show results using multiple instance learning (MILES) and figures on the right side show results using multiple instance learning with discriminative mapping (MILDM).

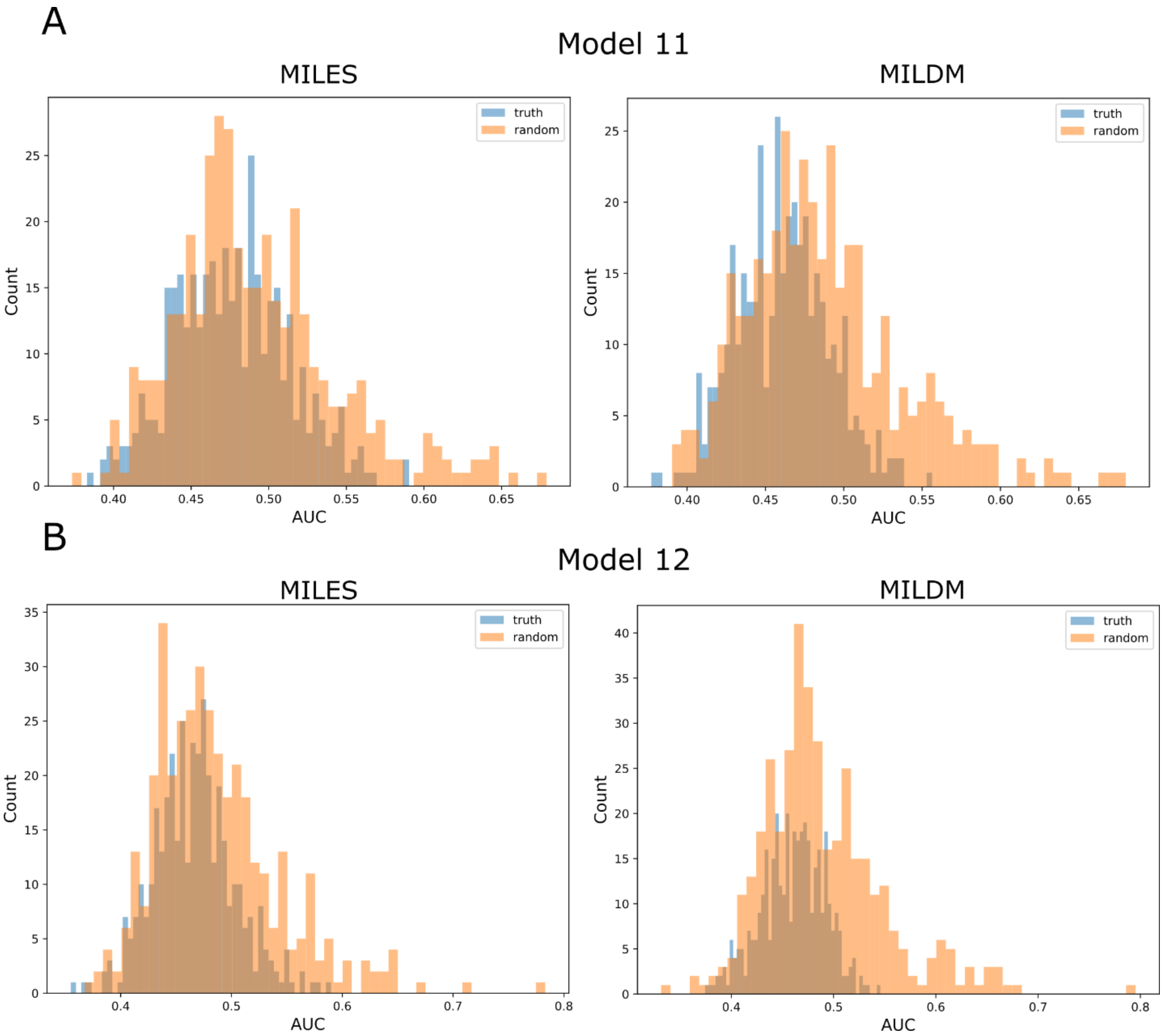

Fig. S12. Histograms plotting each area under curve (AUC) calculated from receiver operating characteristic (ROC) curves for classification models with random and true data labels. 96 classification models were run using random labels (orange bars) compared to true labels (blue bars). These models discriminate between Raman spectra from dried saliva droplets from COVID-negative volunteers based on whether symptoms were classed as respiratory or non-

respiratory taken between 1100 and 1726 cm^{-1}. (A) Model 11 used spectra taken from the "edge" region of a dried droplet from both sexes, (B) Model 12 used spectra from the "on crystal" region. Figures on the left side show results using multiple instance learning (MILES) and figures on the right side show results using multiple instance learning with discriminative mapping (MILDM).

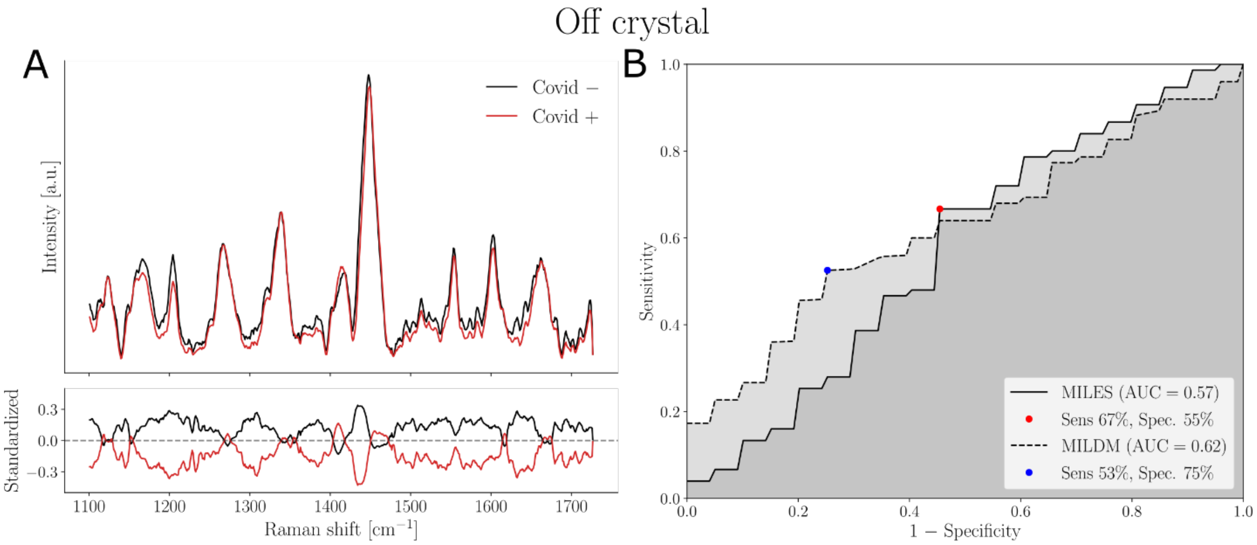

Figure S13:
Machine learning model discriminating between COVID-negative and positive saliva supernatant from males using "off crystal" Raman spectra from dried droplets. (A) Upper frame shows SNV-normalized, baseline corrected Raman spectra from all volunteers. Mean COVID-negative spectra (n = 20, at least 8 spectra per volunteer) are shown in black and COVID-positive spectra (n = 15, at least 8 spectra per volunteer) are shown in red. Bottom frame shows the standardized Raman spectra, where each individual feature has 0 mean and unit variance. (B) Receiver operating curve (ROC) for these models with sensitivity and specificity.

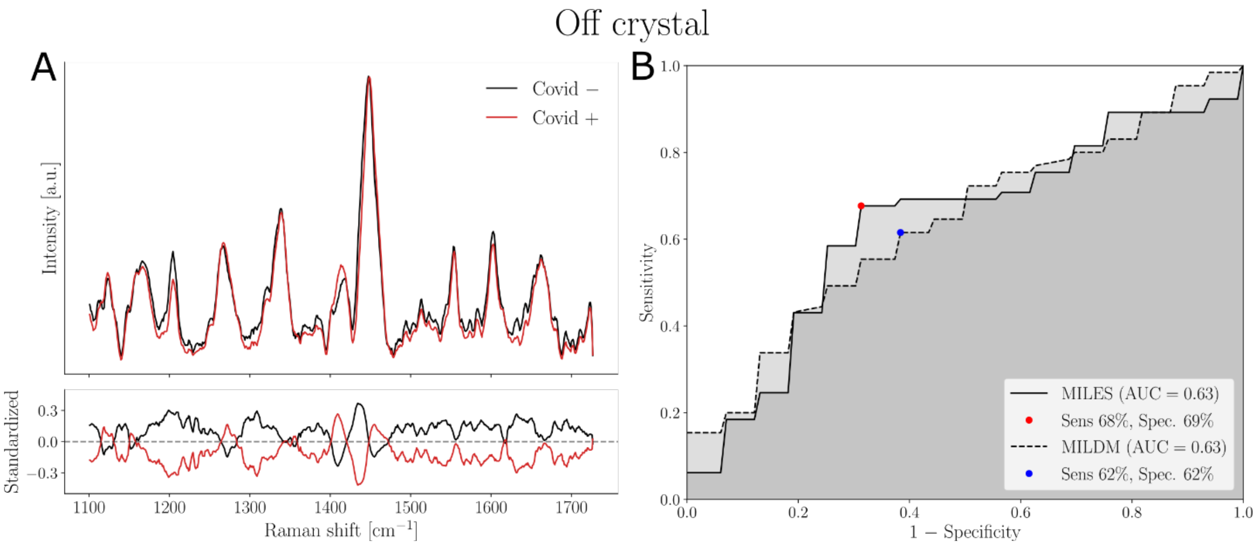

Figure S14:

Machine learning model discriminating between COVID-negative and positive saliva supernatant from females using "off crystal" Raman spectra from dried droplets. (A) Upper frame shows SNV-normalized, baseline corrected Raman spectra from all volunteers. Mean COVID-negative spectra (n = 18, at least 9 spectra per volunteer) are shown in black and COVID-positive spectra (n = 16, at least 9 spectra per volunteer) are shown in red. Bottom frame shows the standardized Raman spectra, where each individual feature has 0 mean and unit variance. (B) Receiver operating curve (ROC) for these models with sensitivity and specificity.

Edge

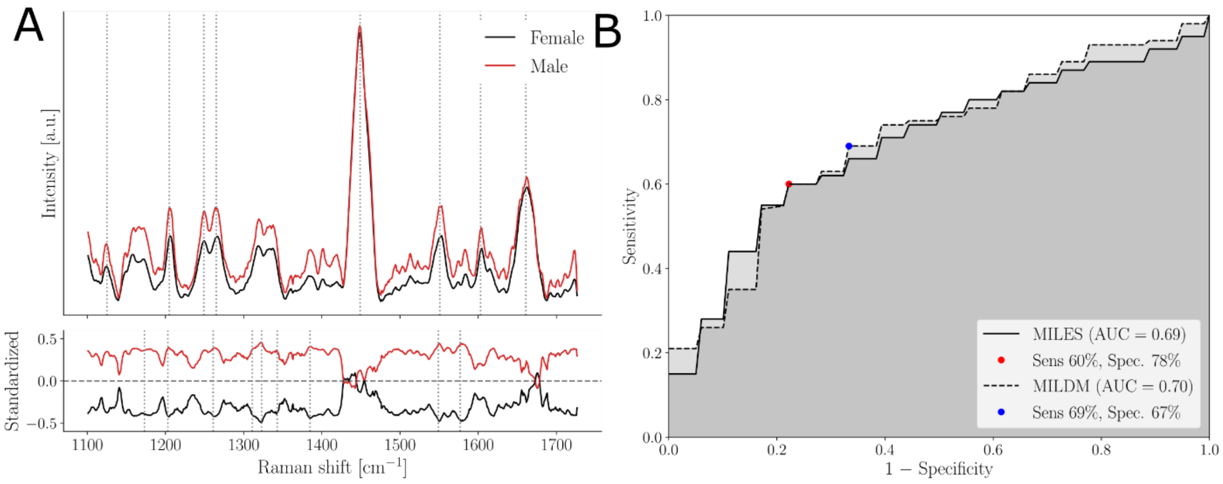

C

Peak center (cm^{-1})	Model saliva correspondence	Biomolecular assignement
1123–1124	Protein	RNA (ribose), lipids, carbohydrates, porphyrin, blood
1203–1206	Protein (amide III)	DNA/RNA, IgG
1248–1249	Protein (amide III), mucin	Asymmetric phosphate, DNA/RNA(guanine, cytosine)
1265–1267	Protein (amide III)	DNA/RNA, fatty acids
1447–1449	Protein (amide I), lactic acid	Lipids, RBCs
1552–1553	Protein(tryptophan), sodium chloride	Mucin, porphyrin
1603–1605	Protein (amide I)	DNA
1661–1663	Protein (amide I)	Unsaturated fatty acids, DNA

On crystal

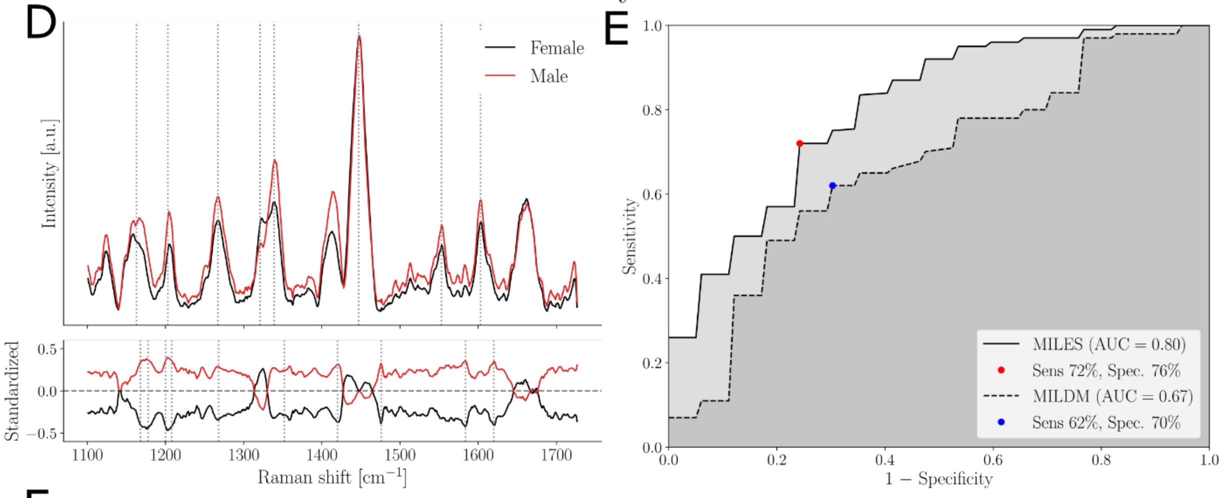

F

Peak center (cm^{-1})	Model saliva correspondence	Biomolecular assignement
1163–1164	Protein	Mucin, quinoid, lipids
1203–1206	Protein (amide III)	DNA/RNA, IgG
1265–1267	Protein (amide III)	DNA/RNA, fatty acids
1321–1323	Protein (amide III)	DNA/RNA (guanine)
1339–1340	Protein	DNA/RNA
1447–1449	Protein (amide I), lactic acid	Lipids, RBCs
1552–1553	Protein(tryptophan), sodium chloride	Mucin, porphyrin
1603–1605	Protein (amide I)	DNA

Fig. S15.
Machine learning model discriminating between female and male saliva supernatant from COVID-negative volunteers using (A-C) “edge” and (D-F) “on crystal” Raman spectra from dried droplets: (A, D) Upper frame shows SNV-normalized, baseline corrected Raman spectra from all volunteers. Variance is shown by pale lines (variance of mean spectrum from each individual) and main features used in model building designated by dotted lines. Mean female spectra (n = 18, at least 9 spectra per volunteer) are shown in black and mean meale spectra (n = 20, at least 9 spectra per volunteer) are shown in red. Bottom frame shows the standardized Raman spectra, where each individual feature has 0 mean and unit variance. (B, E) Receiver operating curve (ROC) for these models with sensitivity and specificity. (C, F) List of features used in model building and their assignments as determined using compounds in model saliva and from literature.

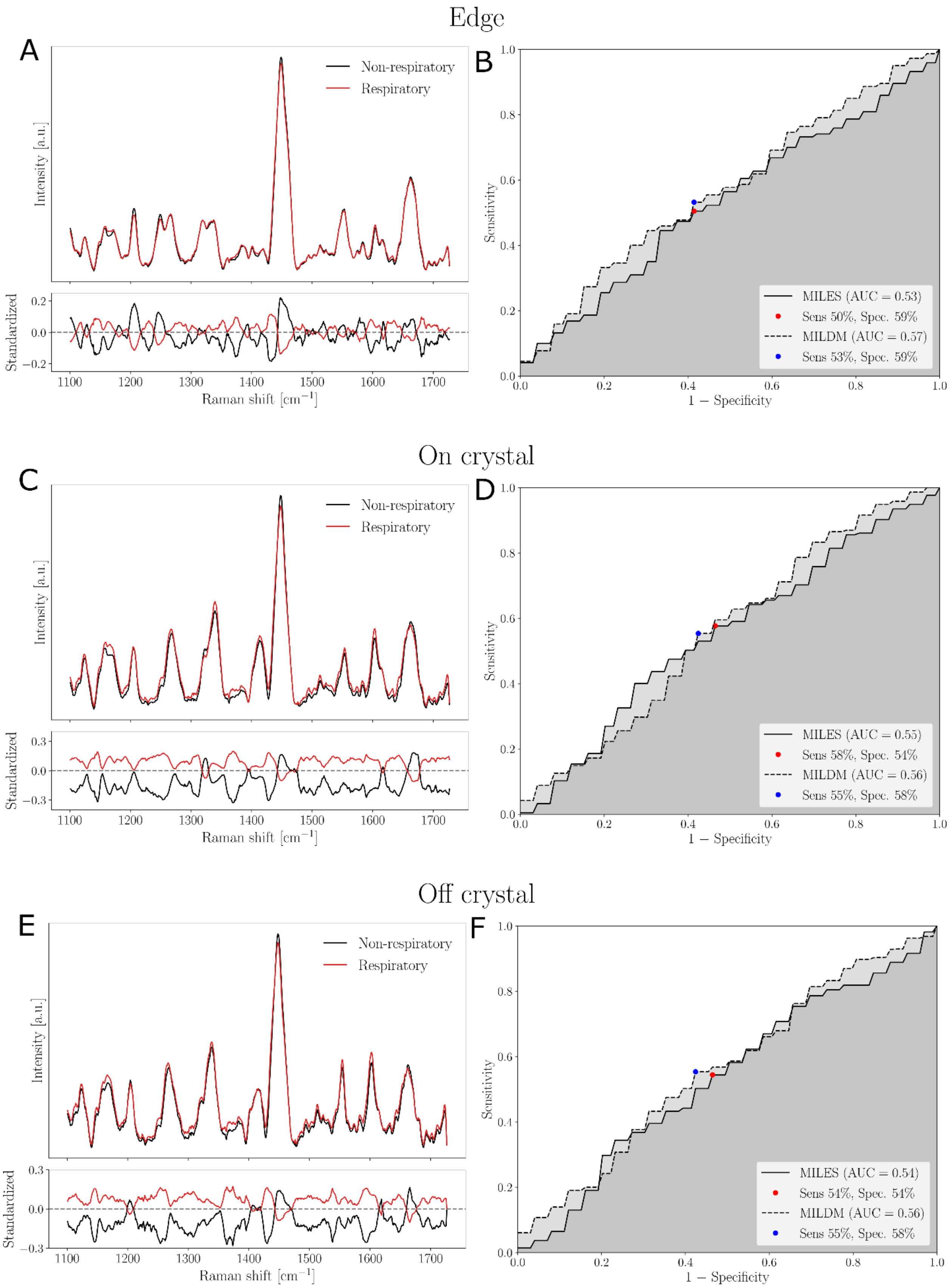
Edge
A
Intensity [a.u.]
Non-respiratory
Respiratory
Standardized
0.2
0.0
−0.2
1100 1200 1300 1400 1500 1600 1700
Raman shift [cm^{-1}]
B
Sensitivity
1 − Specificity
MILES (AUC = 0.53)
Sens 50%, Spec. 59%
MILDM (AUC = 0.57)
Sens 53%, Spec. 59%
On crystal
C
Intensity [a.u.]
Non-respiratory
Respiratory
Standardized
0.3
0.0
−0.3
1100 1200 1300 1400 1500 1600 1700
Raman shift [cm^{-1}]
D
Sensitivity
1 − Specificity
MILES (AUC = 0.55)
Sens 58%, Spec. 54%
MILDM (AUC = 0.56)
Sens 55%, Spec. 58%
Off crystal
E
Intensity [a.u.]
Non-respiratory
Respiratory
Standardized
0.3
0.0
−0.3
1100 1200 1300 1400 1500 1600 1700
Raman shift [cm^{-1}]
F
Sensitivity
1 − Specificity
MILES (AUC = 0.54)
Sens 54%, Spec. 54%
MILDM (AUC = 0.56)
Sens 55%, Spec. 58%
0.0 0.2 0.4 0.6 0.8 1.0

Fig. S16.

Machine learning model discriminating between respiratory and non-respiratory saliva supernatant from volunteers using (A-B) “edge”, (C-D) “on crystal” and (E-F) “off crystal” Raman spectra from dried droplets: (A, C, E) Upper frame shows SNV-normalized, baseline corrected Raman spectra from all volunteers. Variance is shown by pale lines (variance of mean spectrum from each individual) and main features used in model building designated by dotted lines. Mean non-respiratory spectra (n = 23, at least 9 spectra per volunteer) are shown in black and respiratory spectra (n = 44 for edge, 43 for on crystal at least 9 spectra per volunteer) are shown in red. Bottom frame shows the standardized Raman spectra, where each individual feature has 0 mean and unit variance. (B, D, F) Receiver operating curve (ROC) for these models with sensitivity and specificity.
